# Reversible On/Off Switching of Lactide Cyclopolymerization with a Redox-Active Formazanate Ligand

**DOI:** 10.1021/acscatal.1c05689

**Published:** 2022-03-21

**Authors:** Folkert de Vries, Edwin Otten

**Affiliations:** Stratingh Institute for Chemistry, University of Groningen, 9747 AG Groningen, The Netherlands

**Keywords:** catalysis, polymerization, cyclic polylactide, redox-switching, formazanate, redox-active ligand

## Abstract

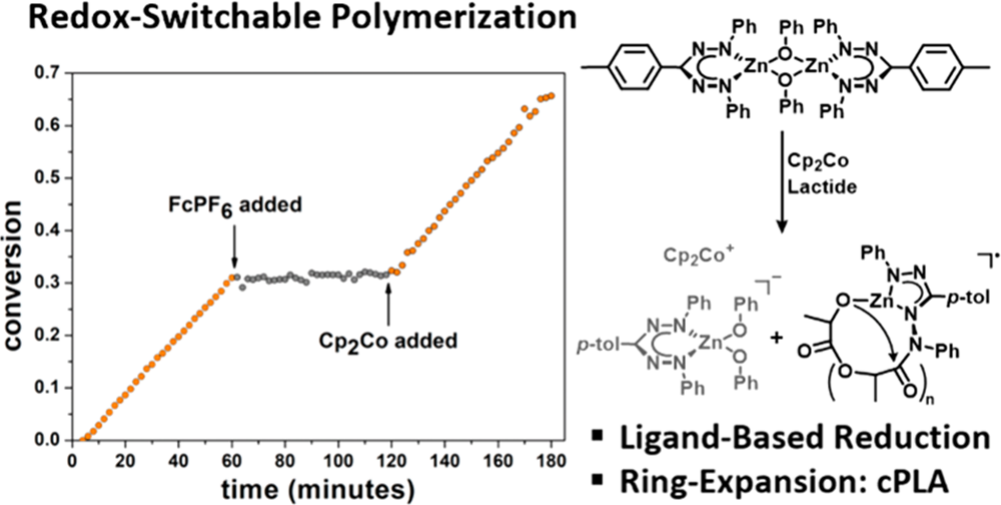

Redox-switching of a formazanate zinc catalyst in ring-opening polymerization (ROP) of lactide is described. Using a redox-active ligand bound to an inert metal ion (Zn^2+^) allows modulation of the catalytic activity by reversible reduction/oxidation chemistry at a purely organic fragment. A combination of kinetic and spectroscopic studies, together with mass spectrometry of the catalysis mixture, provides insight in the nature of the active species and the initiation of lactide ring-opening polymerization. The mechanistic data highlight the key role of the redox-active ligand and provide a rationale for the formation of cyclic polymer.

The synthesis of polymers with tailored properties is made possible by advances in catalysis and postfunctionalization methods to control polymer length, composition, and microstructure. Still, synthetic polymer chemistry pales in comparison to the sophistication achieved in nature’s biopolymers.^1^ Despite remarkable developments in polymerization methods, it has been difficult to exert spatial and/or temporal control, a key characteristic of biological systems to maintain homeostasis. One way to achieve this is by using catalysts that are responsive to electro-, photo-, or mechanochemical stimuli,^2^ providing access to two (or more) states that have distinct reactivity. Wrighton’s pioneering work on redox-switchable Rh hydrogenation catalysis^3^ laid the foundation for the development of complexes that change activity in response to a redox stimulus. In the field of polymerization catalysis, switching of lactide ring-opening polymerization (ROP)^4^ activity via redox chemistry was first described by Gibson and Long (Chart 1a).^5^ This initiated the search for catalysts that provide precise spatiotemporal control of ROP for lactide and other cyclic esters to obtain advanced polymer architectures. An important class of redox-switchable ROP systems is developed by the Diaconescu group using ferrocene-linked chelating ligands (Chart 1b).^6^ In addition to on/off switching, these catalysts also show oxidation-state-dependent monomer selectivity, providing unique block copolymers from a monomer mixture.^7^ Byers et al. developed iron bis(alkoxide) complexes (Chart 1c) that show markedly different reactivity toward cyclic esters in the Fe(II) and Fe(III) states,^8^ and several other redox-switchable ROP catalysts are known.^9−11^ To date, switchable ROP of cyclic esters often relies on metal-based reduction/oxidation either at the “active” central metal or an Fc moiety in the ligand. Redox reactions at nonmetal sites have been much less explored.^12^ Here, we demonstrate an approach that capitalizes on the redox chemistry of a purely organic ligand directly attached to a redox-inactive Zn center. Taking inspiration from the work of Coates and Chisholm on β-diketiminate (BDI) Zn and Mg complexes for lactide ROP,^13,14^ we envisioned that using a formazanate ligand^15^ as a redox-active analogue to the well-known BDIs would impart redox-switching behavior to an otherwise inert catalyst. We demonstrate that this strategy indeed leads to an ROP catalyst that can be switched reversibly between “on” and “off” states using redox chemistry and discuss spectroscopic/kinetic data that corroborates the key role of the formazanate ligand in the switching process as well as the formation of cyclic polylactide (cPLA). Such polymers are of interest due to their distinct physicochemical properties.^16^ To the best of our knowledge, this represents the first example of redox-switchable catalytic synthesis of cPLA.

**Chart 1:**
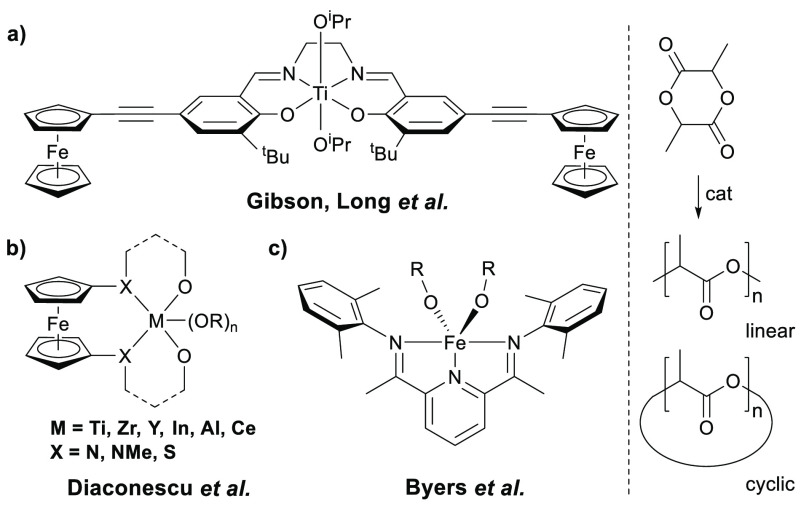
Representative Examples of Catalysts Used in Redox-Switchable Polymerization of Lactide

As a starting point for this research, we prepared the formazanate zinc phenoxide **2** by protonolysis of **1**(17) (Scheme 1). X-ray diffraction revealed that complex **2** exists as a dimer in the solid state, with tetrahedral Zn centers bridged by the phenoxides. Diffusion-ordered NMR spectroscopy in CD_2_Cl_2_ indicates that **2** retains a dimeric structure also in solution.

**Scheme 1:**
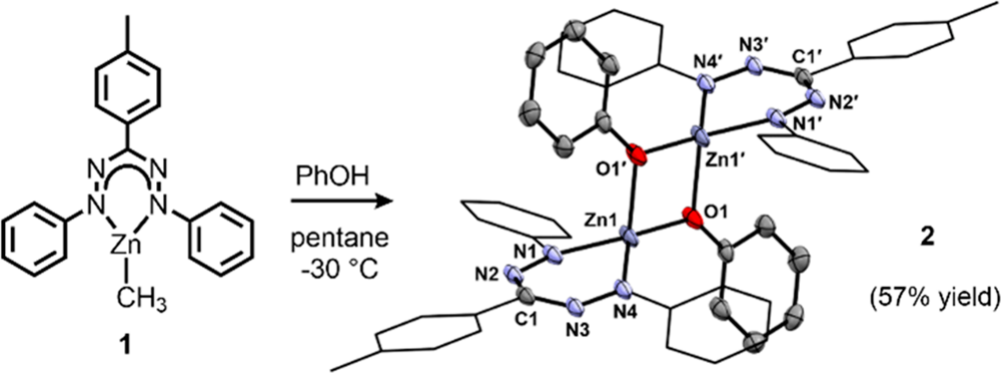
Synthesis of Compound **2** by Protonolysis

The cyclic voltammogram of **2** in THF shows two sequential, closely spaced reductions (*E*_1/2_ ≈ −1.45 and −1.63 V vs Fc^0/+^, Figure S8), which are assigned to the formation of the radical anion **2**^•−^ and the dianion **2**_2−_, respectively. The close spacing and quasireversible nature of both redox couples suggests that all species remain dimeric at least on the time scale of the CV experiment. In CH_2_Cl_2_, a single (overlapping) redox wave is observed with a midpoint potential of ∼ −1.61 V vs Fc^0/+^ (Figure S9).

Turning to ROP catalysis, we found that **2** showed negligible reactivity toward *rac*-lactide (50 equiv) after 12 h in CH_2_Cl_2_ (Table 1, entry 1). This is in stark contrast to related (BDI)Zn catalysts by Coates, which are highly active.^13^ However, addition of the reductant Cp_2_Co to the solution of **2**/lactide (1 equiv of Cp_2_Co per Zn center in **2**; [Co]:[Zn] = 1) turned on ROP activity, and 94% conversion of lactide was reached in 4 h (entry 2). A control experiment with lactide and Cp_2_Co or [Cp_2_Co][PF_6_] in the absence of **2** shows no activity. Thus, Cp_2_Co converts inactive **2** into a reduced species that is catalytically active for lactide ROP. Monitoring the reaction by NMR spectroscopy shows a linear increase in monomer conversion, which indicates a rate law that is zero-order in lactide. It should be noted that metal-catalyzed ROP of cyclic esters is commonly first-order in monomer,^18,13b,19^ but precedent for zero-order behavior exists.^20,21^ The order in catalyst was evaluated by varying the total Zn concentration between 5 and 20 mM (at a constant [Co]:[Zn] ratio of 1). A plot of ln(*k*_obs_) vs ln([Zn]_tot_) afforded a slope of 0.53 (Figure S15). The half-order in [Zn]_tot_ implies that most of the catalyst is present as an inactive dimer, while the active species is monomeric.^22^

**Table 1:** Polymerization of *rac*-Lactide Catalyzed by Formazanate Zinc Alkoxide Complex **2**^a^

entry	[LA]:[Zn]	[Co]:[Zn]	conv. (%)	time (h)	*M*_n_^b^^,^^c^	*M*_w_^b^^,^^c^	*Đ*_M_^d^
1	50:1	-	<1	12	n.d.	n.d.	n.d.
2	50:1	0.5	93	19	12.9	24.6	1.9
3	50:1	1	94	4	6.5	11.4	1.8
4	50:1	2	97	2	5.9	9.5	1.6
5	50:1	4	97	1	4.6	7.2	1.6
6	100:1	1	97	4	11.5	21.0	1.8
7	250:1	1	98	14	14.6	25.4	1.7
8	500:1	1	90	14	17.1	28.6	1.7

a Conditions: [**2**] = 5 mM, 25 °C, 1,3,5-trimethoxybenzene as internal standard, CD_2_Cl_2_ solvent; n.d. = not determined.

b Reported in 10^3^ g mol^–1^.

c Determined by GPC in THF, calibrated versus polystyrene standards.

d *Đ*_M_ = *M*_w_/*M*_n_ (see also ref (23)).

Analysis of the polylactide product (after repeated precipitation from CH_2_Cl_2_/hexane) by NMR spectroscopy did not show the presence of the expected end group (OPh) nor did it contain resonances attributable to the formazan fragment. The MALDI-TOF spectrum contains signals for a polymer with a repeat unit of 72 Da (half a lactide monomer), with a major peak distribution that has the composition of (lactide)_*n*_ + Na^+^(Figure S27). The absence of OPh end groups indicates that in our system the initiation of lactide ROP does *not* occur by nucleophilic attack of the Zn phenoxide; instead, the polymer produced by **2**/Cp_2_Co has a cyclic structure.^16a,16b^ This was corroborated using a derivative of **2** with a Zn–O^*i*^Pr group instead of −OPh, which afforded a polymer with an identical mass spectrum.

The nature of the active species generated from **2** and Cp_2_Co was subject to further investigation. Specifically, it is notable that electrochemical reduction of **2** occurs at a more negative potential than that of Cp_2_Co (*E*_1/2_ = −1.33 vs Fc^0/+^ in CH_2_Cl_2_).^24^ Given the redox potentials, reduction of **2** by Cp_2_Co is likely incomplete when using [Co]:[Zn] = 1. However, in the presence of lactide, the CV shows an additional redox wave at more negative potential, which indicates that (as expected) the reduction product of **2** reacts with lactide (*vide infra*) and shifts the equilibrium. We subsequently monitored the conversion of *rac*-lactide via ^1^H NMR spectroscopy in CD_2_Cl_2_ with increasing [Co]:[Zn] ratios. The result is an approximately linear increase in the reaction rate (Figure 1A), which we ascribe to a higher (equilibrium) concentration of the active catalyst upon increasing the Cp_2_Co concentration. GPC analyses of polymers obtained with different amounts of reductant show that *M*_n_ decreases, and a narrower molecular weight distribution is obtained at higher [Co]:[Zn] ratios (Table 1, entries 2–5), which is in line with an increase in the amount of active catalyst. Doubling the amount of lactide led to an approximate 2-fold increase in *M*_n_ and *M*_w_ (Table 1, entries 3 vs. 6). Further increasing the lactide:[Zn] ratio to 250:1 and 500:1 results in higher molecular weights (entries 7 and 8). The increased viscosity for reaction mixtures with high lactide concentration likely results in mass transfer limitations and lower molecular weights than expected.

**Figure 1:**
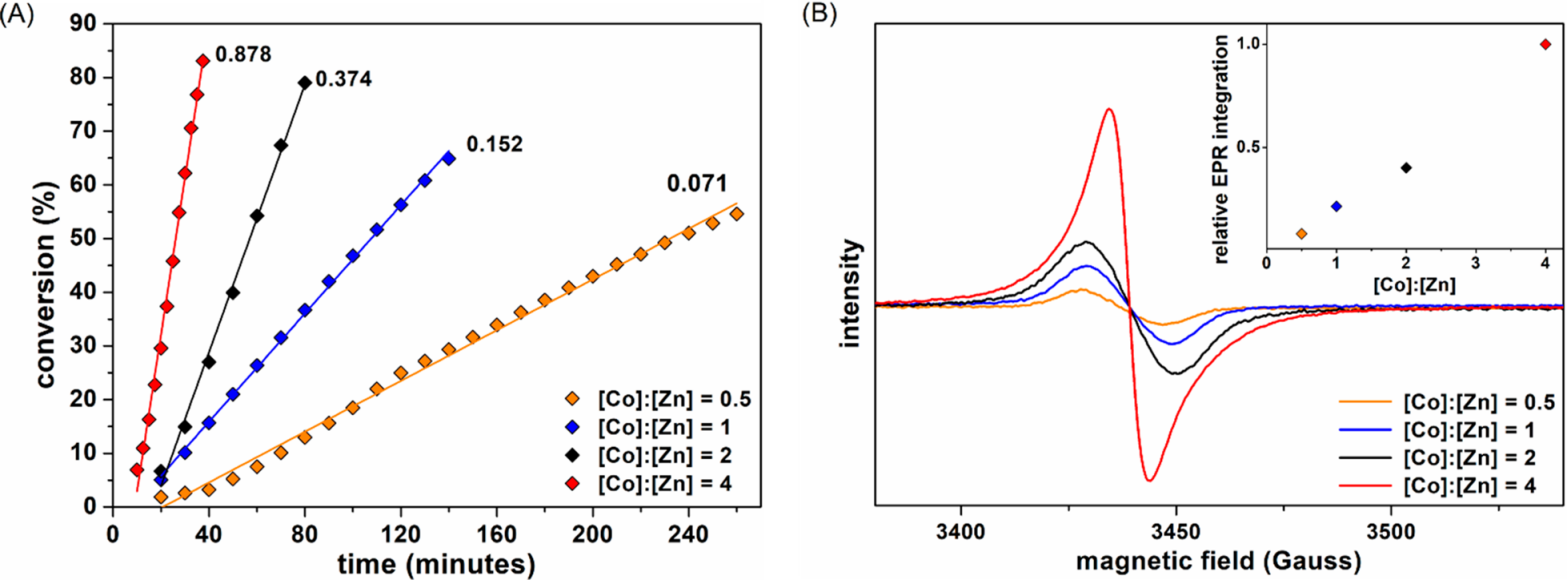
(A) Plot of monomer conversion vs time using **2** with various ratios of [Co]:[Zn] (CD_2_Cl_2_, 25 °C, [LA]_0_/[Zn] = 50, [Zn] = 10 mM). The numbers in the graph correspond to the observed rate constant (M·h^–1^). (B) Room-temperature X-band EPR spectra of **2** (5 mM in CH_2_Cl_2_) with varying amounts of Cp_2_Co added; inset shows the relative double integrations of the EPR spectra with varying [Co]:[Zn] ratios.

EPR spectroscopy provided additional evidence for the proposed redox equilibrium. Data were collected in CH_2_Cl_2_ in the absence of lactide but using the same concentration of **2** and [Co]:[Zn] ratios as in the catalysis experiments.

The room-temperature EPR spectra show a broad signal at *g* ≈ 2 as expected for an organic (ligand) radical (Figure 1B).^25^ Double integration of this signal shows that its intensity increases linearly with the [Co]:[Zn] ratio, in the same way as that the reaction rate increases. To achieve quantitative conversion of **2** to the catalytically active species, we reacted it with the stronger reductant Cp*_2_Co (*E*_1/2_ = −1.94 V vs Fc^0/+^).^24^ Although the catalytic activity of **2**/Cp*_2_Co was indeed generally higher than with Cp_2_Co, the rates were somewhat variable. This may be related to detrimental side reactions with the Cp* Me groups (e.g., CH deprotonation)^26^ as observed in related iron formazanate chemistry.^27^ The UV/vis absorption spectrum of **2** in CH_2_Cl_2_ shows a strong absorption in the visible region (λ_max_ = 549 nm; ε = 33 500 M^–1^ cm^–1^) characteristic for the π–π* transition of formazanate ligands.^17,28^ Addition of either *rac*-lactide, Cp_2_Co, or both results in small changes (see SI), but a low-energy band (λ > 750 nm) characteristic for one-electron reduced formazanate complexes^15^ is either absent or too weak to be observed due to its low equilibrium concentration. Treatment of **2** with Cp*_2_Co does result in appearance of an absorption band at λ ≈ 775 nm (Figure S26), indicating that ligand-based reduction of **2** is indeed feasible. Attempts to isolate the putative reduction product **2**^•−^ were unsuccessful; instead, the salt [Cp*_2_Co]^+^[LZn(OPh)_2_]^−^ (**A**; L = formazanate; Scheme 2) was obtained by crystallization (see SI for the X-ray structure).

**Scheme 2:**
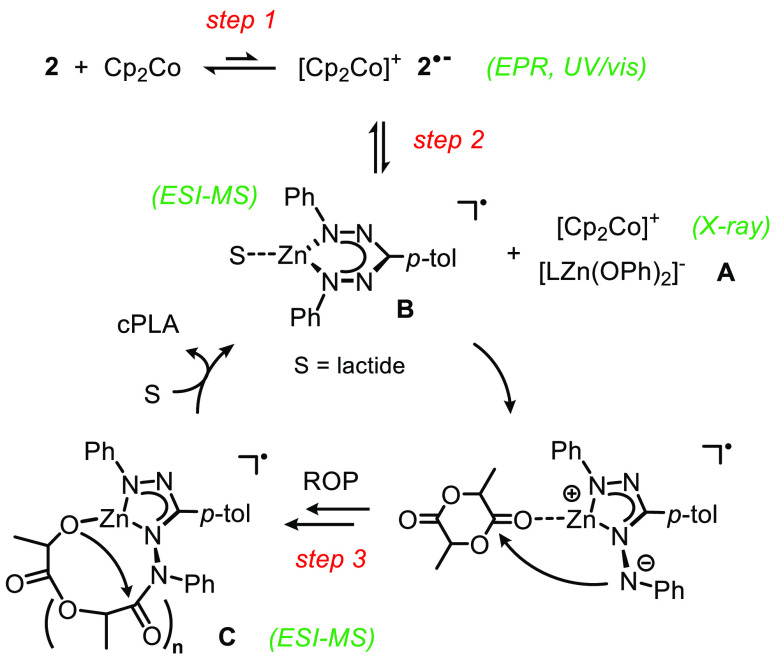
Proposed Mechanism for the Formation of cPLA

Mass spectrometry by direct injection of a polymerization reaction mixture during turnover showed a repeating unit of 72 Da, but different than for the polymers isolated after workup (*vide supra*), we now observe a distribution that indicates an “initiator” of 377.03 Da (Figure S27). This corresponds well with the calculated mass of the (formazanate)Zn fragment (377.07 Da). Taken together, the kinetic and spectroscopic data support the following mechanism for lactide ROP with **2**/Cp_2_Co (Scheme 2). We propose that catalyst activation occurs by ligand-based reduction of the dimer **2** to form the dimeric radical anion **2**^•−^ (step 1). As indicated by the half-order in [Zn], the active catalyst is generated by dissociation into a monomeric Zn species. Rather than breaking up dimer **2**^•−^ in a symmetric fashion, its dissociation into the anion [LZn(OPh)_2_]^−^ (**A**) and neutral L^•^Zn (**B**) (step 2) is supported by crystallography and mass analysis. The isolated zincate **A** (as the Cp*_2_Co^+^ salt) was inactive in lactide ROP, suggesting that **B** is involved in catalysis. The flexible coordination properties of the nitrogen-rich ligand allow liberation of the terminal N atom,^29^ which can attack the coordinated lactide (step 3) to give a ligand-bound growing polymer chain in the form of a macrocyclic intermediate (**C**). Building on the early work of Kricheldorf et al.,^30^ several catalysts are now known to form cyclic polymers via this “ring-expansion” pathway.^16a,16b^

Finally, the reversibility of redox-switching was evaluated. Addition of Cp_2_Co (∼1 equiv per Zn) to a CD_2_Cl_2_ solution of **2** resulted in a substantial shift and broadening of the ^1^H NMR signals of the formazanate ligand (Figure S16). The resonances of the OPh moiety also broaden but remain in the same range (δ 6.4–7.0 ppm). Subsequent addition of 1 equiv of oxidant [Cp_2_Fe][PF_6_] regenerates **2**, demonstrating that the redox chemistry of **2** is chemically reversible. This was corroborated in a catalysis experiment. An NMR tube containing a solution of **2** and 50 equiv of *rac*-lactide was monitored by ^1^H NMR spectroscopy. As expected, no catalytic activity was observed in the absence of reducing agent, but the addition of 1 equiv of Cp_2_Co resulted in a linear increase in conversion (Figure 2).

**Figure 2:**
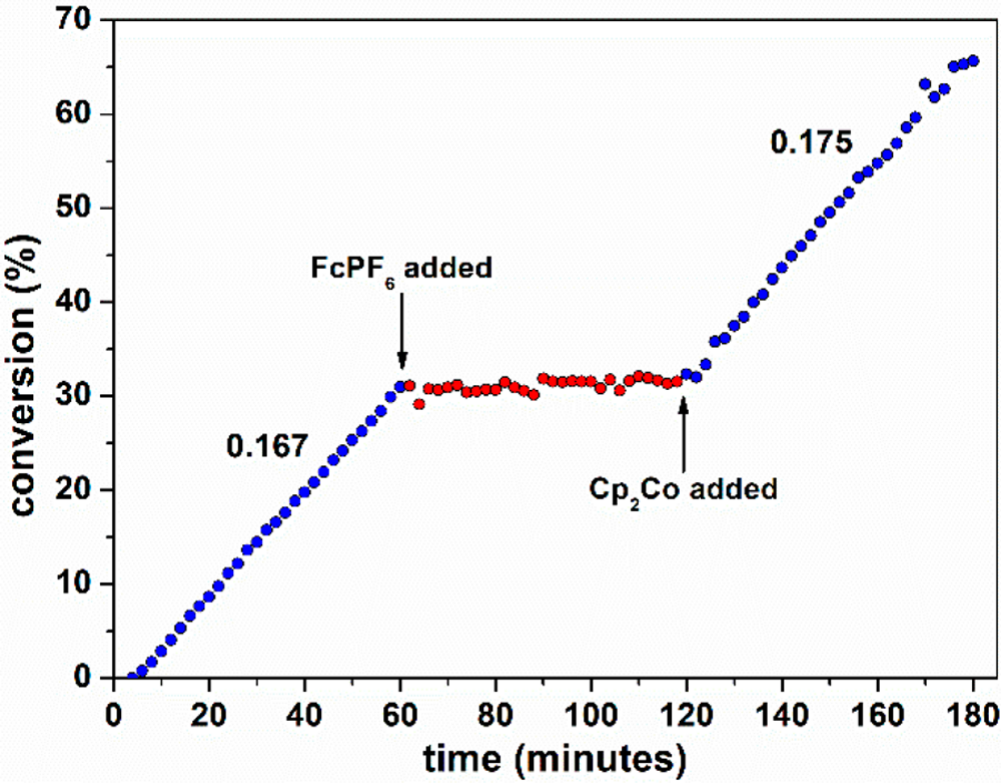
Conversion of lactide monitored by ^1^H NMR spectroscopy (CD_2_Cl_2_, 25 °C, [LA]_0_/[Zn]_0_ = 50, [LA]_0_ = 0.5 M). FcPF_6_ added after 60 min; Cp_2_Co added after 120 min. The numbers indicate the slope of the blue parts of the graph.

After 1 h, a solution containing 1.05 equiv of [Cp_2_Fe][PF_6_] was added, which completely halted the reaction. After another hour, the catalytic activity was fully restored by addition of 1.05 equiv of Cp_2_Co. Thus, switching between “on” and “off” states occurs with excellent reversibility and a high *k*_on_/*k*_off_ ratio.^31^ GPC analysis of samples taken throughout a redox-switching experiment shows that molecular weight increases linearly in the “on” period but is halted when in the “off” state, while dispersities remain low throughout (*Đ*_M_ ≈ 1.2 for conversions < 70%).

In summary, we have demonstrated switchable ring-opening polymerization of *rac*-lactide with a formazanate zinc phenoxide complex. The kinetic and spectroscopic data confirm a key role for the ligand, not only in the redox activation but also as an initiator for the polymerization reaction and formation of cyclic PLA. The ready availability, tunability, and low cost of formazanate ligands make this an attractive platform for development of redox-switchable systems without being limited to the presence of a metal ion as the site of oxidation/reduction, and we anticipate that this could be more broadly applicable to achieve spatiotemporal control also for other catalytic reactions.

## Supporting Information Available

Experimental procedures and characterization data (PDF)
Crystallographic data for **A** (CIF)
Crystallographic data for **2** (CIF)

## Supplementary Material

cs1c05689_si_001.pdf

cs1c05689_si_002.cif

cs1c05689_si_003.cif
