# Supplementary material for: Reversible On/Off Switching of Lactide Cyclopolymerization with a Redox-Active Formazanate Ligand
Source: ACS Catal. 2022 Mar 21;12(7):4125–30. doi: 10.1021/acscatal.1c05689 (PMC8981207; doi:10.1021/acscatal.1c05689)
Supplement: Supplementary file 1 — cs1c05689_si_001.pdf [file cs1c05689_si_001.pdf]

Supporting Information to

Reversible On/Off Switching of Lactide Cyclopolymerization with Redox-Active Formazanate Ligand

Folkert de Vries, Edwin Otten*

Stratingh Institute for Chemistry, University of Groningen

9747 AG Groningen, The Netherlands

* email: edwin.otten@rug.nl

Table of Contents

General Considerations 2

Experimentals 3

Comparison of Representative Redox-Switchable Catalysts for Lactide Polymerization 5

NMR Spectroscopy 6

X-ray Crystallography 8

Cyclic Voltammetry 12

NMR Scale Experiments 15

EPR Spectroscopy 18

UV-Vis Spectroscopy 20

Direct Injection Mass Spectrometry 23

Characterization of the Resulting Polymers 24

References 28

General Considerations

All manipulations, except for ligand synthesis, were carried out under nitrogen atmosphere using glovebox, Schlenk and vacuum-line techniques. Glassware was dried before use at 150 °C. Ligand **L1H**[1] and complex **1**[2] were synthesized via literature procedures. The reagents used for the synthesis of ligand **L1H** and complex **1** were used as received; *p*-tolualdehyde (Sigma-Aldrich, 97%), phenylhydrazine (Sigma-Aldrich, 97%), aniline (Sigma-Aldrich, 99%), sodium nitrite (Sigma-Aldrich, 99%), hydrochloric acid (Boom B.V., 37–38%), dimethylzinc (ABCR, 10 w% in hexane), phenol (Merck, 99%). *Rac*-lactide (Sigma-Aldrich) was recrystallized three times from dry toluene and dried on a vacuum pump. Toluene and pentane (Sigma-Aldrich, anhydrous, 99.8%) were passed over columns of Al_2O_3 (Fluka), BASF R3-11-supported Cu oxygen scavenger, and molecular sieves (Sigma-Aldrich, 4 Å). Deuterated solvents were vacuum transferred from CaH_2 (CD_2Cl_2, Euriso-top) and stored under nitrogen.

NMR spectra were recorded on Varian Mercury Plus 400, Varian Inova 500 or Bruker Avance NEO 600 spectrometers. The ^1H and ^{13}C NMR spectra were referenced internally using the residual solvent resonances and reported in ppm relative to TMS (0 ppm). All electrochemical measurements were performed at ambient temperatures under an inert N_2 atmosphere in THF or DCM, containing 0.1 M $[Bu_4N][PF_6]$ as the supporting electrolyte. Electrochemical measurements were performed using an Autolab PGSTAT 204 computer-controlled potentiostat and data was recorded with Autolab NOVA software (v.2.1.4). Cyclic voltammetry (CV) was performed using a three-electrode configuration comprising of a Pt wire counter electrode, a Ag wire pseudo-reference electrode and a Pt disk working electrode (CHI102, CH Instruments, diameter = 2 mm). The Pt working electrode was polished before the experiment using an alumina slurry (0.05 µm), rinsed with distilled water and subjected to brief ultrasonication to remove any adhered alumina microparticles. The electrodes were then dried in an oven at 75 °C overnight to remove any residual traces of water. The CV data was referenced by addition of ferrocene to the THF or DCM solution at the end of experiments. UV/Vis spectra were recorded in a DCM solution (≈ 10^{-5} M) using an Avantes AvaSpec-2048 UV/Vis spectrophotometer. Electron paramagnetic resonance (EPR) measurements were carried out on a Bruker EMX Nano X-band (9.5 GHz) and performed under nitrogen with a sample concentration of 1 mM in degassed dichloromethane. Polymer samples for GPC and MALDI-TOF measurements were obtained by quenching the reaction with a droplet of MeOH followed by precipitation of the polymer from a DCM solution, by addition to excess hexane (3x) and the precipitated polymer was subsequently dried under vacuum. Molecular weights (M_n) and (M_w) and the dispersity ($Đ_M$) were measured by gel permeation chromatography (GPC) using triple detection, consisting of a Viscotek RALLS detector, Viscotek Viscometer Model H502 and Schambeck RI2012,A Refractive Index detector. The separation was carried out by utilizing two PLgel 5 µm MIXED-C, 300 mm columns from Agilent Technologies at 35 °C. THF 99+%, extra pure, stabilized with BHT was used as the eluent at a flow rate of 1.0 mL/min. The samples were filtered over a 0.2 µm PTFE filter prior to injection. Data acquisition and calculations were performed using Viscotek OmniSec software version 5.0, using a refractive index increment (dn/dc) of 0.042. Molecular weights were determined based on a universal calibration curve generated from narrow dispersity polystyrene standards (Agilent and Polymer Laboratories, *M*w from 645 to 3001000 g/mol). MALDI-TOF mass spectra were recorded on an AB Sciex 4800 *Plus* MALDI-TOF/TOF Analyzer. solutions of DHB as a matrix (20 mg/mL in THF), polymer sample (5 mg/mL in THF) and LiCl (5 mg/mL in THF) were prepared. MALDI-TOF samples were prepared by mixing the before-mentioned solutions in a matrix:sample:salt = 10:2:1 ratio. 1 µL of this solution was applied onto the MALDI target plate. Mass spectrometry by direct injection was performed on a Waters Xevo G2 QTOF spectrometer using ESI, measuring in negative ionization mode.

Experimentals

1,5-diphenyl-3-*para*-tolyl formazanate zinc phenoxide (complex 2)

1,5-diphenyl-3-*para*-tolyl formazanate zinc methyl (325 mg, 0.825 mmol) was dissolved in 15 mL of toluene. The solution was layered with pentane (10 mL) containing phenol (77.0 mg, 0.818 mmol) and was left overnight to diffuse in a freezer at -30 °C. Crystals had formed overnight which were separated from the solution, washed with toluene (2 times, 2 mL) and pentane (7 times, 2 mL) and dried *in vacuo*, to afforded 245 mg (0.236 mmol, 57 %) of large crystals with a green metallic shine. The crystals obtained were suitable for X-ray diffraction. ^{1}H-NMR (400 MHz, CD_2Cl_2, 25 °C): δ 8.06 (d, 4H, *p*-Tol *o*-CH), 7.82 (d, 8H, Ph *o*-CH), 7.33 (d, 4H, *p*-Tol *m*-CH), 7.24 (t, toluene)*, 7.15 (m, toluene)*, 7.12 (m, 4H, Ph *p*-CH), 7.07 (m, 8H, Ph *m*-CH), 6.93 (t, 4H, PhO *m*-CH), 6.66 (t, 2H, PhO *p*-CH), 6.44 (d, 4H, PhO *o*-CH), 2.45 (s, 6H, *p*-Tol *p*-CH_3), 2.34 (s, toluene)* ppm. ^{13}C-NMR (151 MHz, CD_2Cl_2, 25 °C): δ 158.9 (PhO *ipso*-C), 152.8 (Ph *ipso*-C), 143.2 (NN*C*NN), 137.7 (pTol *ipso*-C), 136.9 (p-Tol *p*-C), 130.4 (PhO *m*-CH), 130.1 (Ph *m*-CH), 129.6 (pTol *m*-CH), 129.4 (toluene *o*-CH)*, 128.6 (toluene *m*-CH)*, 128.4 (Ph *p*-CH), 126.2 (pTol *o*-CH), 125.6 (toluene *p*-CH)*, 120.7 (Ph *o*-CH), 120.1 (PhO *p*-CH), 118.0 (PhO *o*-CH), 21.6 (toluene CH_3)*, 21.4 (*p*-Tol *p*-CH_3) ppm. *Crystals of **2** contain one molecule of toluene per dimer based on sc-XRD. Anal. Calcd for $C_{52}H_{44}N_8O_2Zn_2 \cdot C_7H_8$: C 68.41, H 5.06, N 10.82; found: C 68.37, H 5.06, N 10.69.

Typical NMR scale polymerizations

In a glove box, *rac*-lactide (250 µmol), 1,3,5-trimethoxybenzene (25 µmol) and catalyst **2** (2.5 µmol) were added to a vial and dissolved in 0.4 mL CD_2Cl_2 and subsequently transferred to a screw-cap NMR tube, fitted with a PTFE/silicone septum. A solution of Cp_2Co (5.0 µmol, 1 eq. per formazanate, in 100 µL CD_2Cl_2) was prepared and kept in a 100 µL micro-syringe fitted with a rubber stopper. The sample tube was removed from the glove box and a ^{1}H NMR spectrum was measured on a Varian Inova 500 spectrometer prior to 'activating' the catalyst. The Cp_2Co solution (5.0 µmol, 1 eq. per formazanate, in 100 µL CD_2Cl_2) was added through the septum using a micro-syringe, which initiated the polymerization. Data was collected automatically using an arrayed experiment with a pre-acquisition delay ("pad"). Conversion was determined on the basis of the ^{1}H NMR integrations of methine peak of LA and PLA versus the integration of the aromatic signal of the internal standard TMB.

Procedure for NMR scale polymerizations with different equivalents of Cp_2Co

In a glove box, *rac*-lactide (250 µmol), 1,3,5-trimethoxybenzene (25 µmol) and catalyst **2** (2.5 µmol) were added to a vial and dissolved in 0.4 mL CD_2Cl_2 and subsequently transferred to a screw-cap NMR tube, fitted with a PTFE/silicone septum. Solutions with different concentration of Cp_2Co were prepared and kept in a 100 µL micro-syringe fitted with a rubber stopper.* The sample tube was removed from the glove box and a ^{1}H NMR spectrum was measured on a Varian Inova 500 prior to 'activating' the catalyst. The Cp_2Co solution was added through the septum using a micro-syringe, which initiated the polymerization.

*A larger amount of CD_2Cl_2 was needed to dissolve the 4 equivalents of Cp_2Co, this resulted in the use of 250 µL micro-syringe. The amount of CD_2Cl_2 used to dissolve the *rac*-lactide, 1,3,5-trimethoxybenzene and catalyst was scaled down, so that the total volume after addition amounted to 500 µL. Due to the smaller amount of solvent, no spectrum was recorded prior to activation of the catalyst.

Procedure for NMR scale switching studies

In a glove box, *rac*-lactide (250 µmol), 1,3,5-trimethoxybenzene (25 µmol) and catalyst **1** (2.5 µmol) were added to a vial and dissolved in 0.4 mL CD_2Cl_2 and subsequently transferred to a screw-cap NMR tube, fitted with a PTFE/silicone septum. Solutions of Cp_2Co and $FcPF_6$ were prepared and kept in a 100 µL micro-syringe fitted with a rubber stopper. The sample tube was removed from the glove box and a ^1H NMR spectrum was measured on a Varian Inova 500 prior to 'activating' the catalyst. The Cp_2Co solution (5.0 µmol in 100 µL CD_2Cl_2, [Co]:[Zn] = 1.0) was added through the septum using a micro-syringe, which initiated the polymerization. Data was collected automatically every 2 minutes for a total of 60 minutes (using the array "pad" command). The sample tube was removed from the instrument, $FcPF_6$ solution (5.2 µmol in 100 µL CD_2Cl_2, [Fe]:[Zn] ~ 1.05) was added through the septum and the tube was given a good shake to mix everything thoroughly. The sample was placed back in the instrument and measurements were continued. After another 60 minutes, the same procedure was repeated with a Cp_2Co solution (5.2 µmol in 100 µL CD_2Cl_2, [Co]:[Zn] ~ 1.05) to reactivate the catalyst. The polymerization was monitored to approximately 70% conversion. Conversion was determined on the basis of the ^1H NMR integrations of methine peak of LA and PLA versus the integration of the aromatic signal of the internal standard TMB.

Procedure for the NMR scale stability of the OFF-state

In a glove box, *rac*-lactide (250 µmol) and catalyst **2** (2.5 µmol) were added to a vial and dissolved in 0.4 mL CD_2Cl_2 and subsequently transferred to a J. Young NMR tube. A Cp_2Co solution (5.0 µmol in 100 µL CD_2Cl_2, [Co]:[Zn] = 1.0) was added using a micro-syringe, which initiated the polymerization, after which the NMR tube was taken out of the glove box and placed in the NMR (Varian Inova 500) and spectra were collected automatically every 5 minutes up to 60 minutes after initiation (using the array "pad" command). The sample tube was removed from the instrument, taken into the glove box, $FcPF_6$ solution (5.2 µmol in 100 µL CD_2Cl_2, [Fe]:[Zn] ~ 1.05) was added and the tube was given a good shake to mix everything thoroughly, after which another spectrum was measured. The sample was stored in the glove box overnight, and the next morning a spectrum was measured (still in the OFF-state) after a total of 18 hours. After 17 hours in the OFF-state, Cp_2Co solution (5.2 µmol in 100 µL CD_2Cl_2, [Co]:[Zn] ~ 1.05) was added to the tube (in glove box) to reactivate the catalyst. The polymerization was monitored to approximately 95% conversion.

Procedure for the M_n versus conversion plot

In a glove box, *rac*-lactide (2.5 mmol) and catalyst (12.5 µmol) were dissolved in 5 mL DCM. The polymerization was initiated by the addition of Cp_2Co (25 µmol, [Co]:[Zn] = 1.0) and aliquots (250 µL) were taken every 15 minutes. These aliquots were quenched by addition of a $FcPF_6$ solution, taken out of the glove box and evaporated. After 45 minutes, $FcPF_6$ (20 µmol, [Fe]:[Zn] = 1.0) was added to the reaction mixture, halting the polymerization. After another 45 minutes (total time 90 minutes), the reaction was restarted by the addition of Cp_2Co (17 µmol, [Co]:[Zn] = 1.05). Conversion was determined on the basis of the ^1H NMR integrations and the number average molecular weight (M_n) and dispersity were measured by GPC analysis.

Polymerizations with higher LA loading

In a glove box, *rac*-lactide (0.625 or 1.25 mmol) and catalyst (2.5 µmol) were dissolved in DCM (to yield a 0.5 M solution in LA). The polymerization was initiated by the addition of Cp_2Co (5 µmol, [Co]:[Zn] = 1.0) and after 14 hours, aliquots were taken to assess the conversion. The reaction was worked up when conversion had reached > 90%. The polymers were purified by precipitated in hexane (3x).

Comparison of Representative Redox-Switchable Catalysts for Lactide Polymerization

Table S1.

Catalyst	M_n	Đ	LA equiv.	Rate constant (h^{-1}) [a]	# of Switches [b]	Reference
**A**	6.5	1.2 – 1.8[c]	100	0.15 M h^{-1} [d]	2	This work
**B**	13.5	1.14	100	0.0018	1	3
**C**	11.1	1.34	100	1.17	3	4
**D**	7.83	1.10	100	0.92	3	5
**D**[e] **(Echem)**	6.9	1.0	100	0.43	1[f]	6
**E**	9.6	1.09	50	0.54	3	7
**E**[e] **(Echem)**	9.3	1.24	100	0.12	1[g]	8
**F**	11.5	1.20	100	0.23	1[h]	9
**G**	8.0	1.18	100	0.80	2	10

[a] Converted to h^{-1} from the data presented in the reference/ESI. [b] One switch defined as switching reactivity OFF and back ON again. [c] The dispersity of the polymer broadens significantly upon standing after conversion of all the lactide, intrinsic dispersities of ≈ 1.2 were observed at lower conversion of LA (Figure S33). [d] Polymerization is zero order in LA, therefore different units apply. [e] Switches were performed by electrochemical methods. [f] One switch for the homopolymerization of LA, two switches performed in the copolymerization with CHO. [g] Catalyst switched ON at the start, plus one regular switch. [h] First switch induces a change in ligand coordination geometry, the subsequent switch allows for modulation of the activity in the ROP of lactide.

(A)

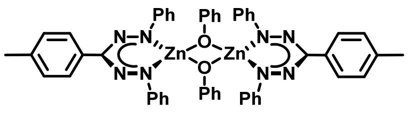

(B)

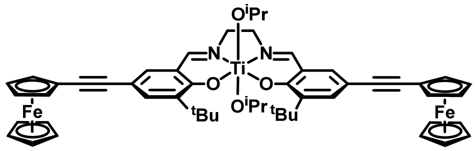

(C)

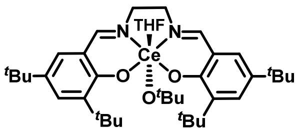

(D)

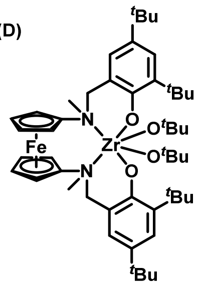

(E)

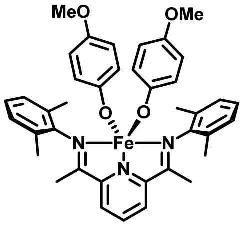

(F)

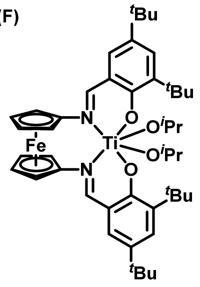

(G)

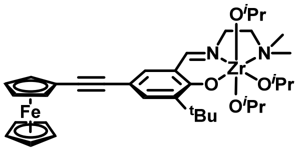

NMR Spectroscopy

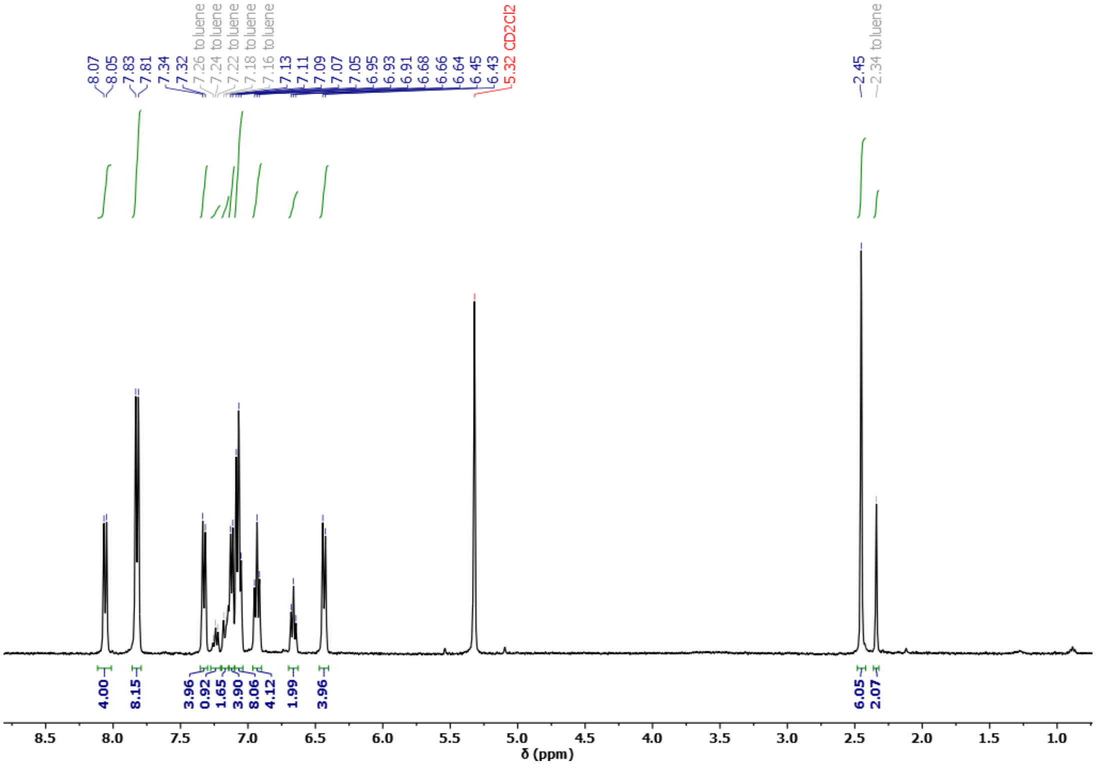

Figure S1. ^{1}H NMR spectrum of **2** (CD_2Cl_2, 25 °C, 400 MHz).

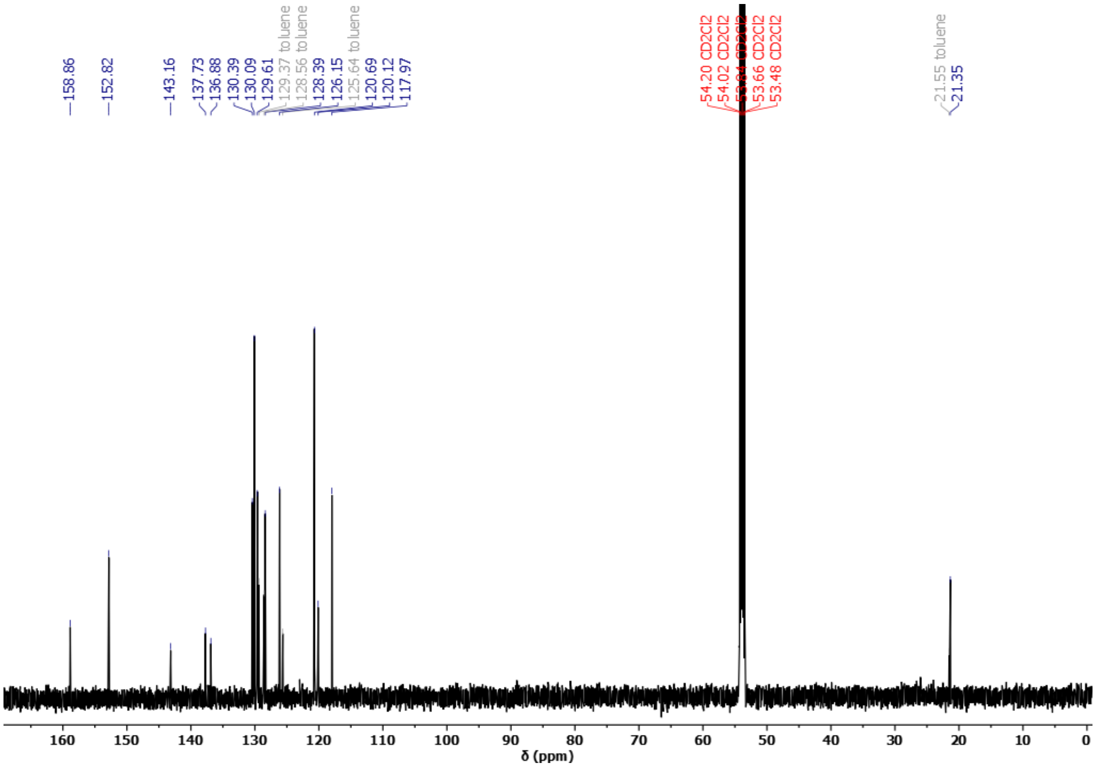

Figure S2. ^{13}C NMR spectrum of **2** (CD_2Cl_2, 25 °C, 151 MHz).

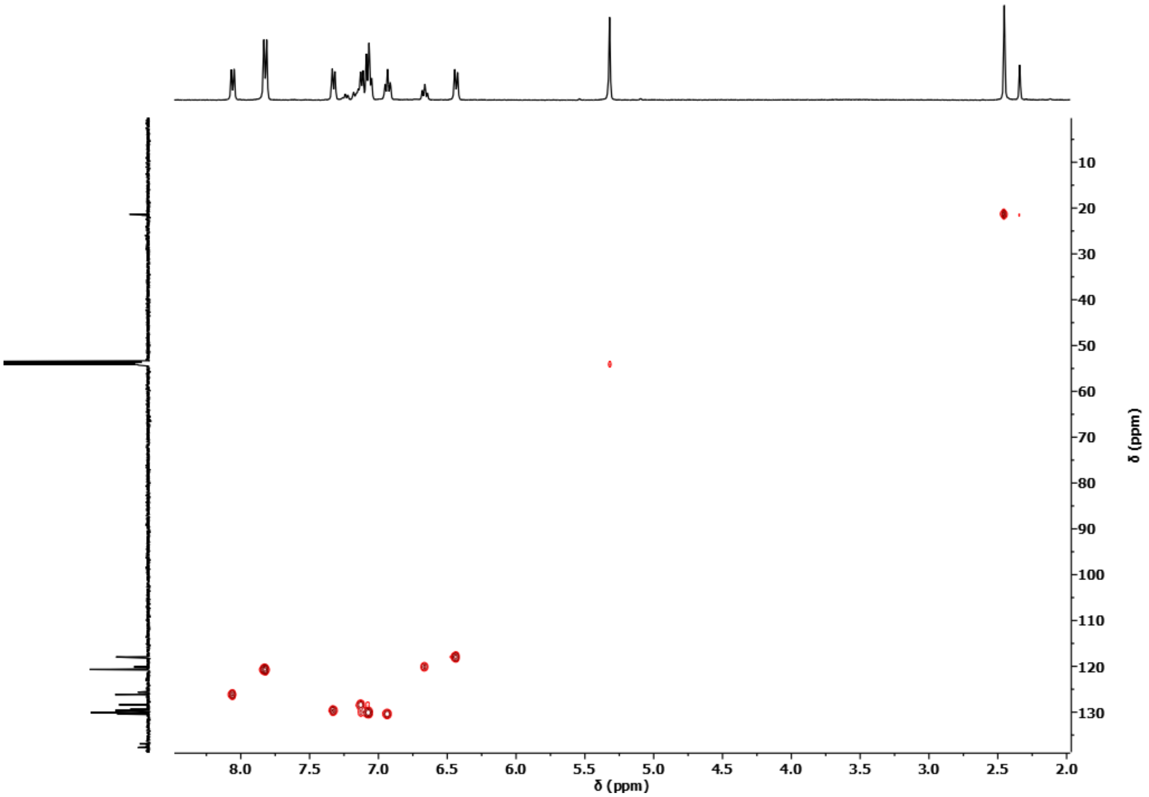

Figure S3. ^{1}H-^{13}C gHSQC spectrum of **2** (CD_2Cl_2, 25 °C, 600 MHz).

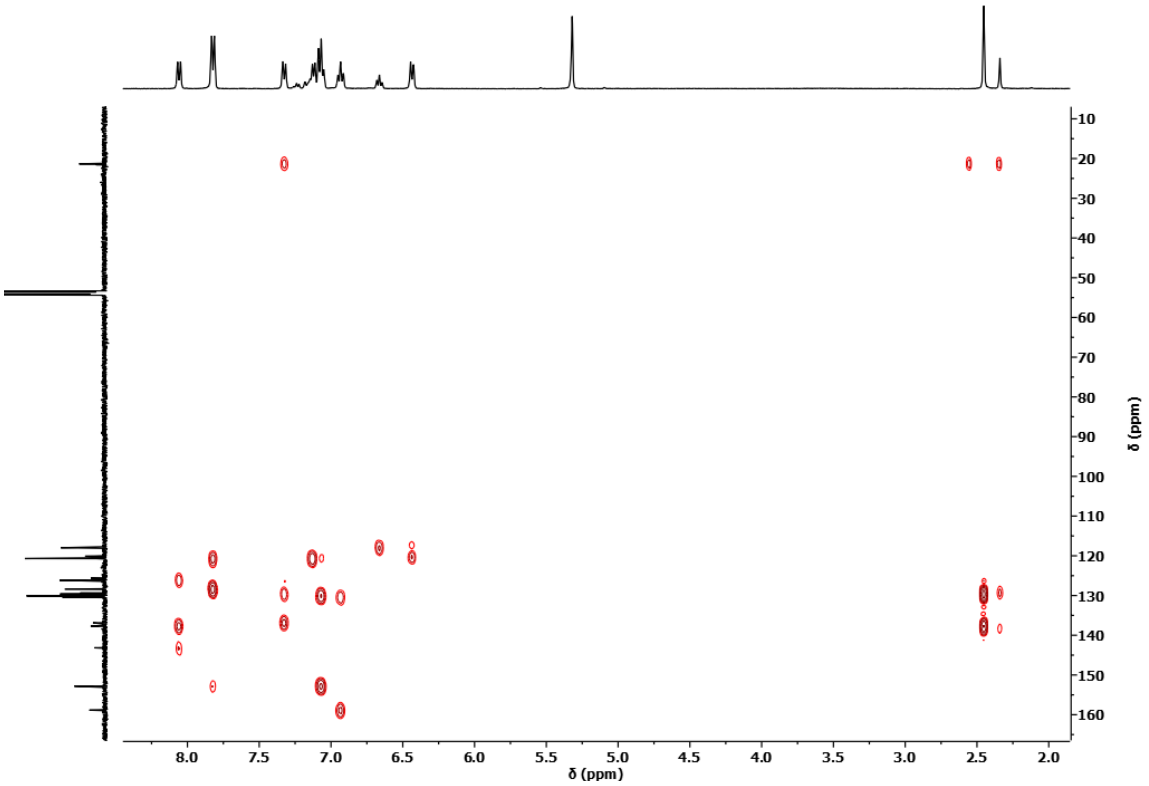

Figure S4. ^{1}H-^{13}C gHMBC spectrum of **2** (CD_2Cl_2, 25 °C, 600 MHz).

X-ray Crystallography

Suitable crystals of **2** and $[LZn(OPh)_2][Cp^*{}_2Co]$ (**A**) were mounted on a cryo-loop in a glove box and transferred, using inert atmosphere handling techniques, into the cold nitrogen stream of a Bruker D8 Venture diffractometer. Data collection and reduction was done using the Bruker software suite APEX3.[11] The final unit cell was obtained from the xyz centroids of 9902 (**2**), 9994 (**A**), reflections after integration. A multi-scan absorption correction was applied, based on the intensities of symmetry-related reflections measured at different angular settings (SADABS).[11] The structure was solved by dual space methods using the program SHELXT.[12] Structure refinement was performed with the program package SHELXL.[13] The hydrogen atoms were generated by geometrical considerations and constrained to idealized geometries and allowed to ride on their carrier atoms with an isotropic displacement parameter related to the equivalent displacement parameter of their carrier atoms. For complex **2**, the toluene solvent molecule was disordered over an inversion center. The symmetry was disregarded in the refinement (negative PART number) and its phenyl ring was constrained to be a regular hexagon (AFIX 66). Three reflections with (I_{obs}/I_{calc})/Sigma(W) > 10 were omitted from the final refinement. For compound **A**, initial refinement yielded the atom positions of the molecules of interest and one CH_2Cl_2 solvent molecule. However, an area with high residual electron density was present in the difference Fourier map, which indicated the presence of highly disordered solvent molecules. The contribution from this area was removed by a PLATON/SQUEEZE[14] run which identified a solvent accessible void containing 42 e^- (possibly a pentane molecule), subsequent refinement proceeded smoothly. Crystal data and details on data collection and refinement are presented in Table S2.

Table S2. Crystallographic data for compound **2** and **A**.

	**2**	**A**
chem formula	$C_{59}H_{52}N_8O_2Zn_2$	$C_{53}H_{59}Cl_2CoN_4O_2Zn$
M_r	1035.82	979.24
cryst syst	triclinic	triclinic
color, habit	dark blue, block	dark red, diamond
size (mm)	0.423 x 0.200 x 0.060	0.209 x 0.129 x 0.092
space group	P-1	P-1
a (Å)	9.1508(3)	10.7552(3)
b (Å)	10.8692(4)	11.5789(3)
c (Å)	14.2352(5)	21.0514(5)
α (°)	80.7300(10)	103.4640(10)
β (°)	73.2480(10)	93.1180(10)
γ (°)	67.2520(10)	103.0100(10)
V (Å^3)	1248.34(8)	2468.27(11)
Z	1	2
ρ_{calc}, g.cm^{-3}	1.378	1.318
Radiation [Å]	Cu Kα 1.54178	Cu Kα 1.54178
μ(Cu K_α), mm^{-1}	1.595	4.560
F(000)	538	1024
temp (K)	100	100
θ range (°)	3.247 – 72.562	2.172 – 74.586
data collected (h,k,l)	-11:10; -13:13; -17:17	-13:13; -14:13; -26:26
no. of rflns collected	31685	90473
no. of indpndt reflns	4939	10076
observed reflns $F_o \geq 2.0\ \sigma\ (F_o)$	4715	9421
R(F) (%)	2.70	3.76
wR(F^2) (%)	6.81	9.84
GooF	1.043	1.038
weighting a,b	0.0243, 0.7544	0.0483, 1.8592
params refined	342	579
min, max resid dens	-0.656, 0.288	-0.875, 0.781

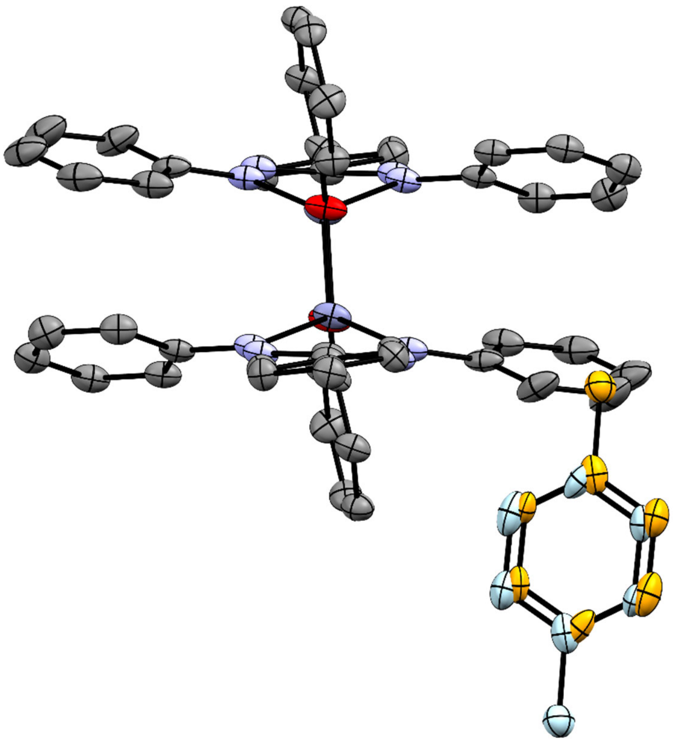

Figure S5. Molecular structure of **2**, showing the disordered toluene molecule. Hydrogen atoms omitted for clarity.

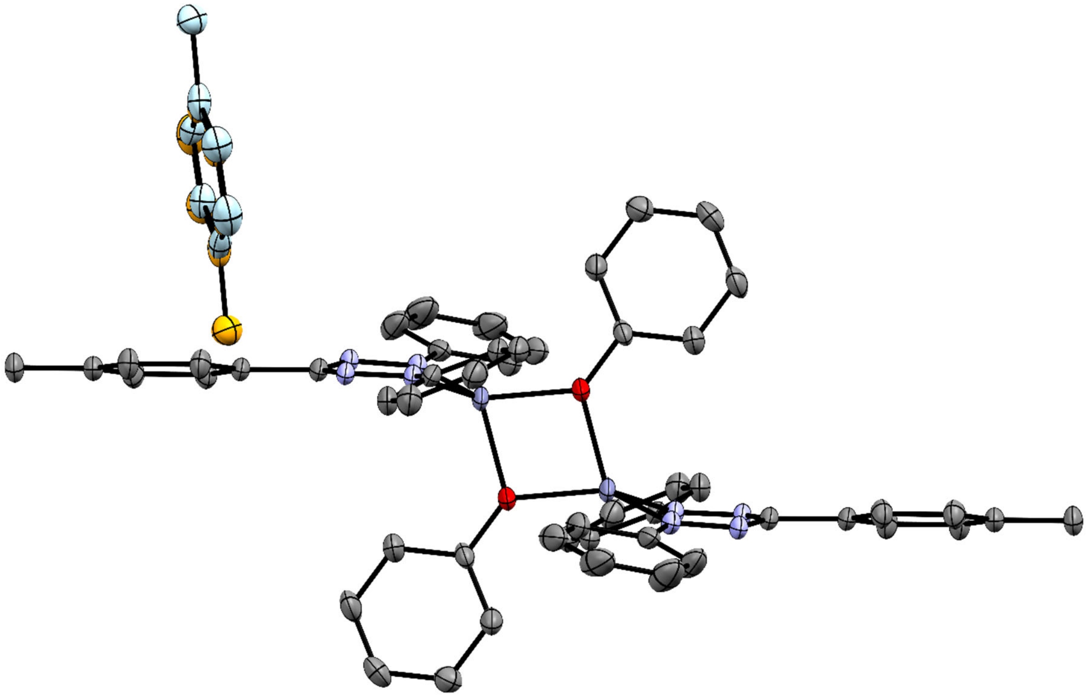

Figure S6. Molecular structure of **2**, showing the side view of the dimeric formazanate zinc phenoxide complex. Hydrogen atoms omitted for clarity.

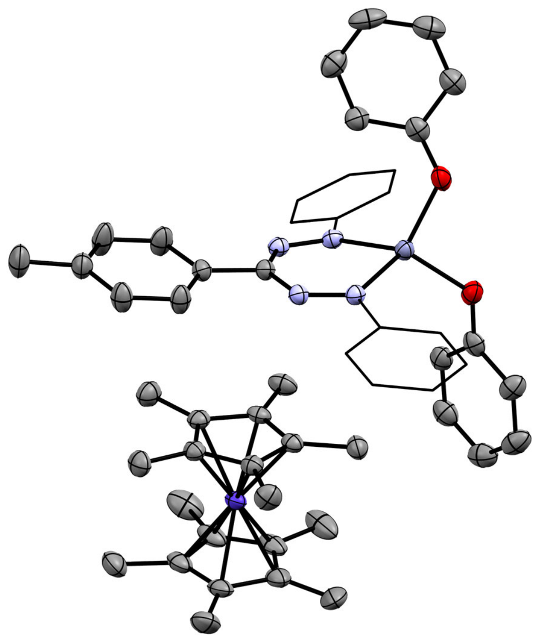

Figure S7. Molecular structure of $[LZn(OPh)_2][Cp^*{}_2Co]$ (**A**), hydrogen atoms and DCM solvent molecule omitted for clarity.

Cyclic Voltammetry

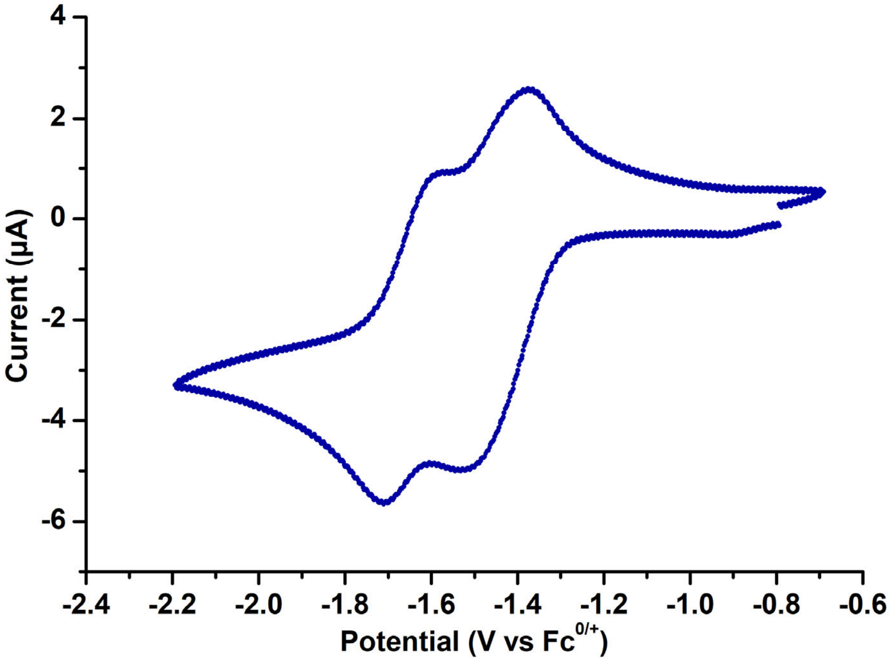

Figure S8. Cyclic voltammogram of **2** (THF, 0.1 M [Bu_4N][PF_6] electrolyte solution) at a scan rate of 100 mV/s.

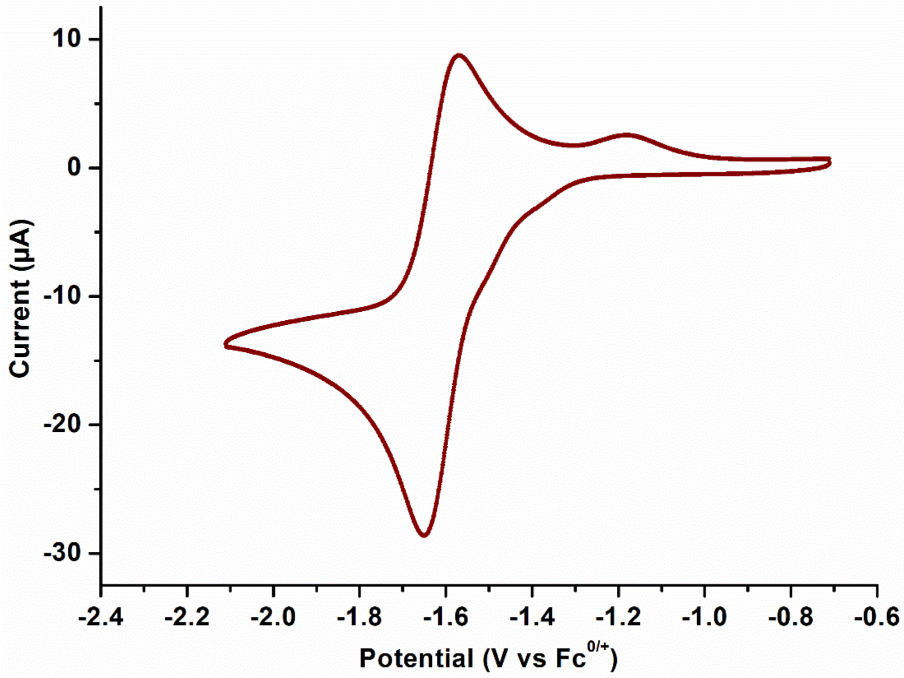

Figure S9. Cyclic voltammogram of **2** (CH_2Cl_2, 0.1 M [Bu_4N][PF_6] electrolyte solution) at a scan rate of 100 mV/s.

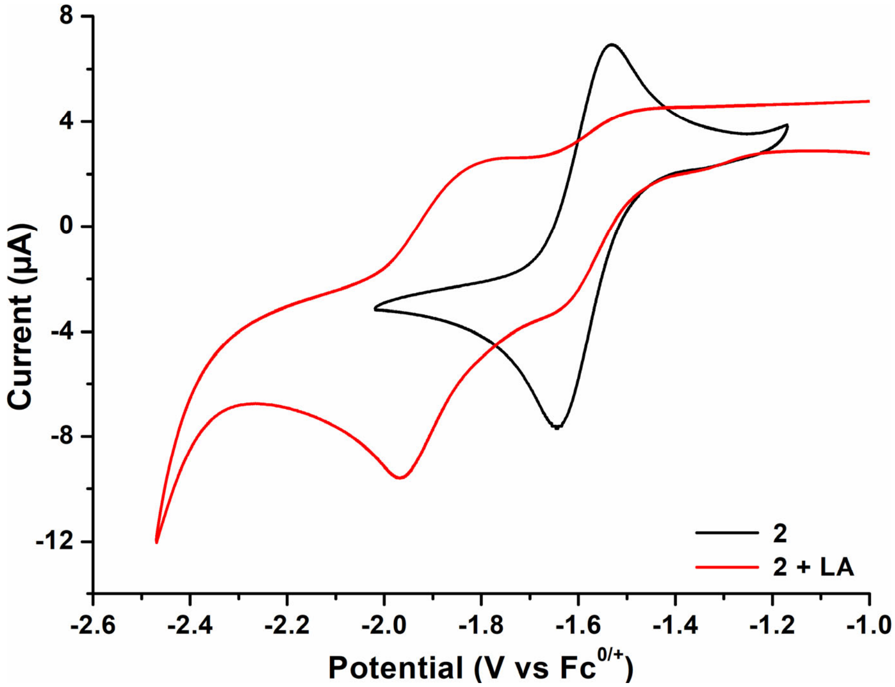

Figure S10. Cyclic voltammogram of **2**, with and without lactide (50 equiv.) present (CH_2Cl_2, 0.1 M $[Bu_4N][PF_6]$ electrolyte solution) at a scan rate of 100 mV/s.

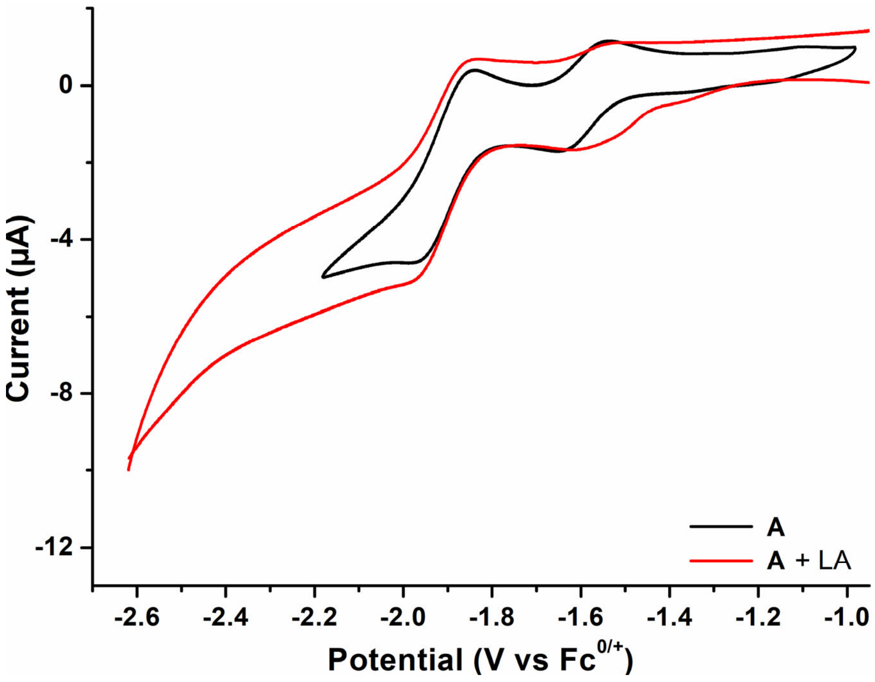

Figure S11. Cyclic voltammogram of **A**, with and without lactide (50 equiv.) present (CH_2Cl_2, 0.1 M $[Bu_4N][PF_6]$ electrolyte solution) at a scan rate of 100 mV/s.

DOSY NMR Spectroscopy

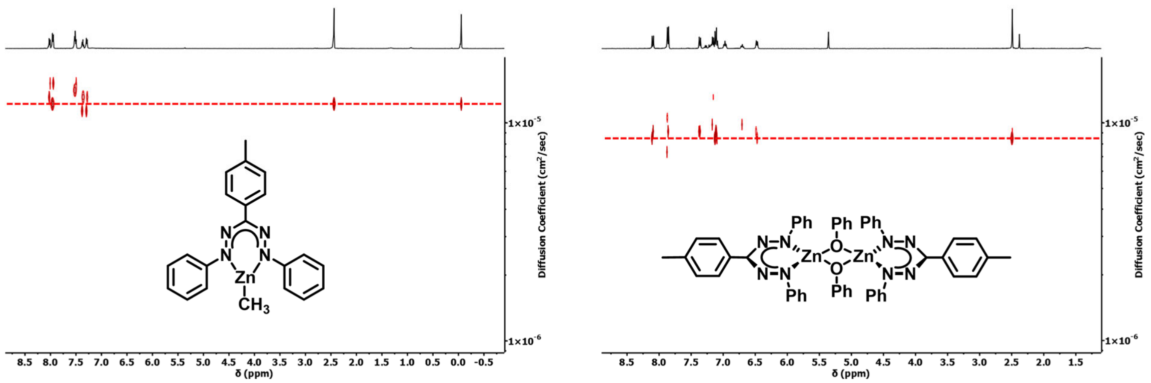

Figure S12. ccDOSY NMR spectra of **1** (left) and **2** (right) (CD_2Cl_2, 25 °C, 500 MHz).

To evaluate whether **2** would retain its dimeric structure in solution, ccDOSY (convection compensated) was performed on both compound **1** and **2**. Compound **1** was set as the benchmark for the approximate size (r) for the monomeric species. The hydrodynamic radius of **2** calculated from the DOSY data is significantly larger than the one found for **1**, from which we conclude that that **2** remains dimeric in solution.

$$D = \frac{k_B T}{6\pi\eta r} \quad \textbf{(Equation S1)}$$

Table S3. Parameters obtained from the DOSY NMR experiment.

Compound	Diffusion coefficient (m^2/sec)[a]	r (Å) [b]
**1**	12.4×10^{-10}	4.26
**2**	8.46×10^{-10}	6.25

[a] Obtained from DOSY NMR experiment. [b] Calculated using the Stokes-Einstein equation (Equation S1).

NMR Scale Experiments

NMR Scale Kinetics

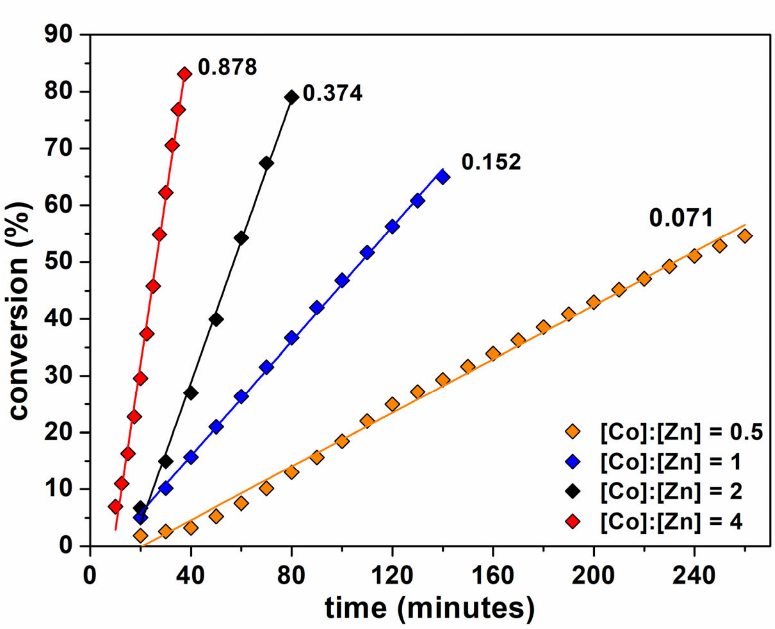

[Co]:[Zn] = 0.5

Equation	y = a + b*x		
Weight	No Weighting		
Residual Sum of Squares	0.00339		
Adj. R-Square	0.99515		
		Value	Standard Error
D	Intercept	-0.04877	0.00531
	Slope	0.00236	3.36906E-5

[Co]:[Zn] = 1

Equation	y = a + b*x		
Weight	No Weighting		
Residual Sum of Squares	5.59682E-4		
Adj. R-Square	0.99868		
		Value	Standard Error
B	Intercept	-0.04282	0.00467
	Slope	0.00505	5.28736E-5

[Co]:[Zn] = 2

Equation	y = a + b*x		
Weight	No Weighting		
Residual Sum of Squares	0.00166		
Adj. R-Square	0.99545		
		Value	Standard Error
F	Intercept	-0.21036	0.01852
	Slope	0.01247	3.43907E-4

[Co]:[Zn] = 4

Equation	y = a + b*x		
Weight	No Weighting		
Residual Sum of Squares	0.00399		
Adj. R-Square	0.9943		
		Value	Standard Error
H	Intercept	-0.26355	0.01688
	Slope	0.02925	6.67821E-4

Figure S13. Plot of monomer conversion *vs* time using **2** with different [Co]:[Zn] ratios (CD_2Cl_2, 25 °C, $[LA]_0/[Zn]_0$ = 50, $[Zn]_0$ = 10 mM). Tables on the right of the plot give the statistics of the linear fit. The numbers in the plot correspond to the observed zeroth-order rate constant ($M \cdot h^{-1}$).

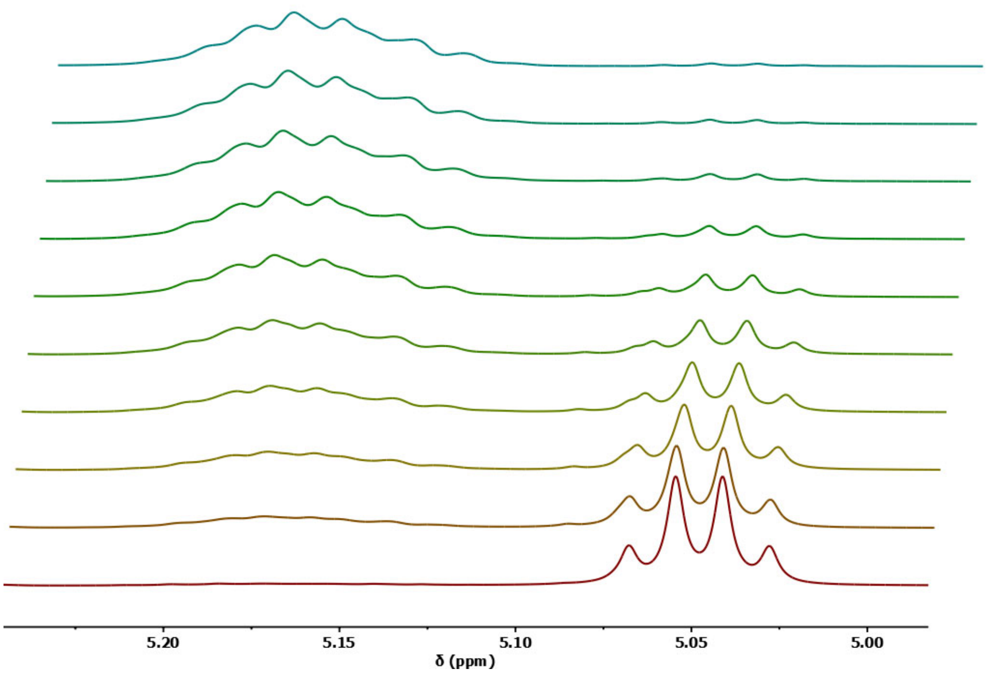

Figure S14. Representative NMR spectra ([Co]:[Zn] = 2) of the conversion of LA to PLA, focusing on the methine proton region. Time interval between spectra is 10 minutes (CD_2Cl_2, 25 °C, 500 MHz).

Order in Catalyst

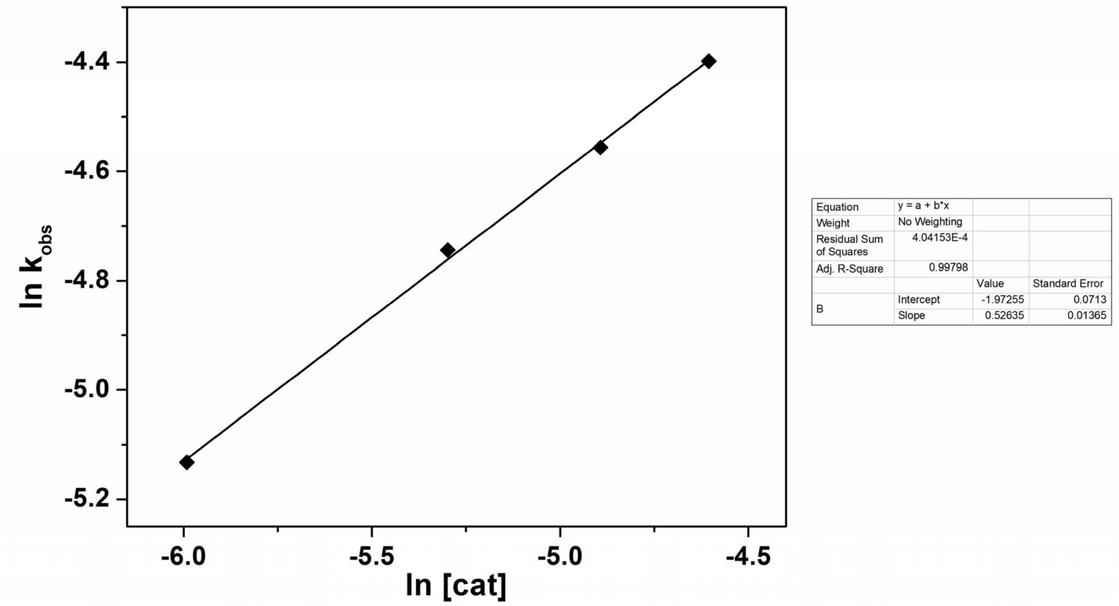

Figure S15. Plot of ln(k_{obs}) *vs* ln([cat]) for the polymerization of *rac*-lactide with **2** (CD_2Cl_2, 25 °C, $[LA]_0$ = 0.5 M, [Co]:[Zn] = 1).

NMR Scale Reduction

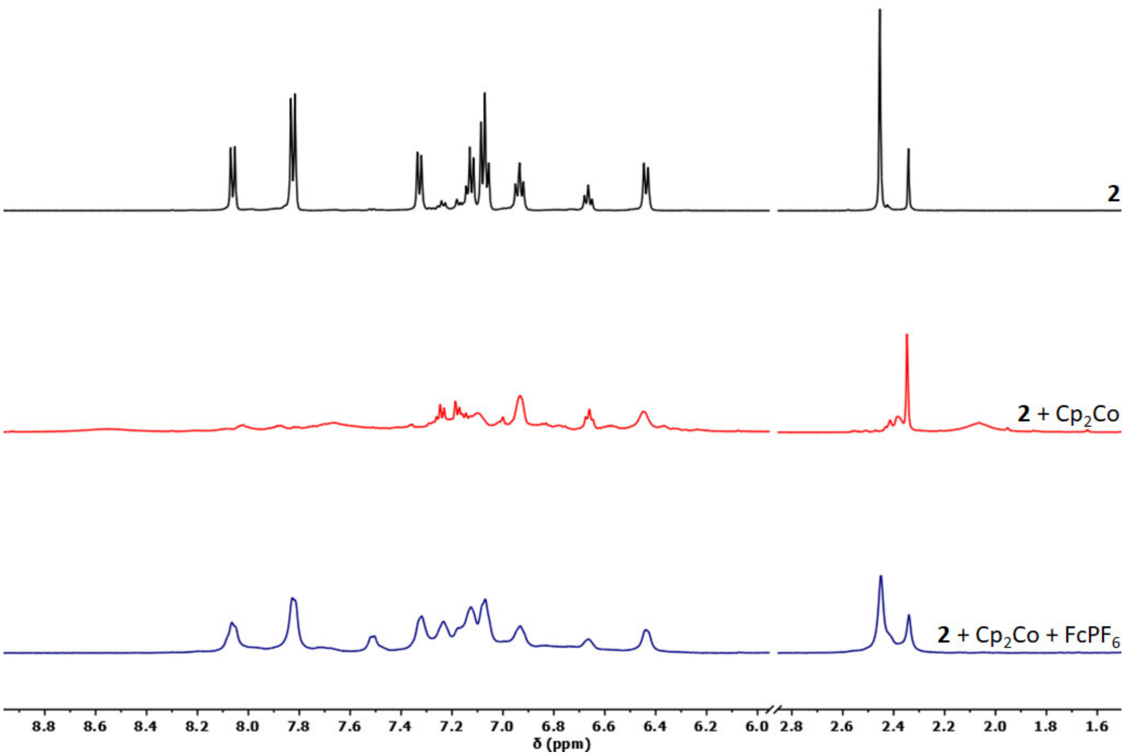

Figure S16. ^{1}H NMR spectra of **2** (top), **2** + Cp_2Co (middle) and **2** + Cp_2Co + $FcPF_6$ (bottom) (CD_2Cl_2, 25 °C, 500 MHz).

NMR Scale Switching Studies

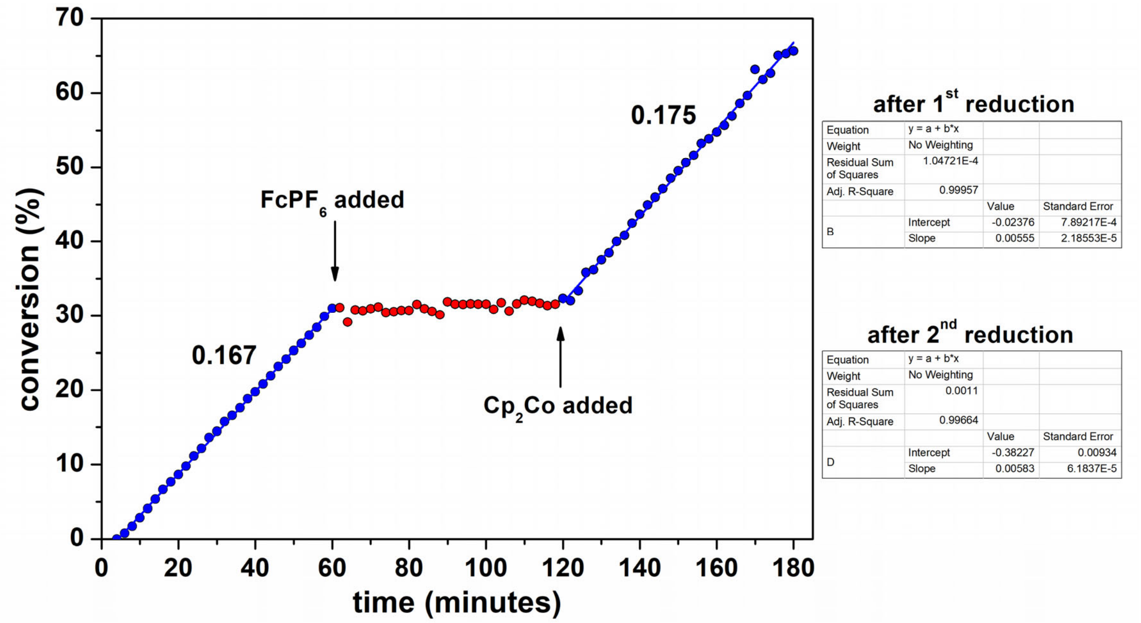

Figure S17. Conversion of lactide as monitored by ^1H NMR (CD_2Cl_2, 25 °C, $[LA]_0/[Zn]_0 = 50$, $[LA]_0 = 0.5$ M). $FcPF_6$ added after 60 minutes, Cp_2Co added after 120 minutes. Tables on the right of the plot give the statistics of the linear fit. The numbers in the plot correspond to the observed zeroth-order rate constant ($M \cdot h^{-1}$).

Stability of the OFF-state

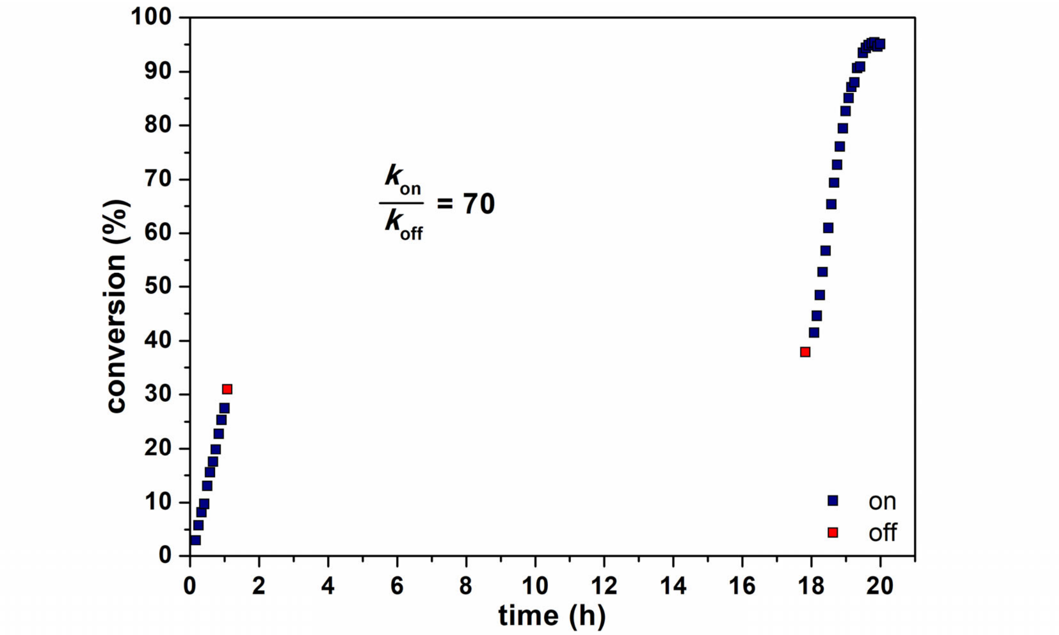

Figure S18. Conversion of lactide over time with the catalyst kept in the OFF-state for 17 hours, monitored by ^1H NMR (CD_2Cl_2, 25 °C, $[LA]_0/[Zn]_0 = 50$, $[LA]_0 = 0.5$ M). $FcPF_6$ added after 1 hour, Cp_2Co added after 18 hours.

EPR Spectroscopy

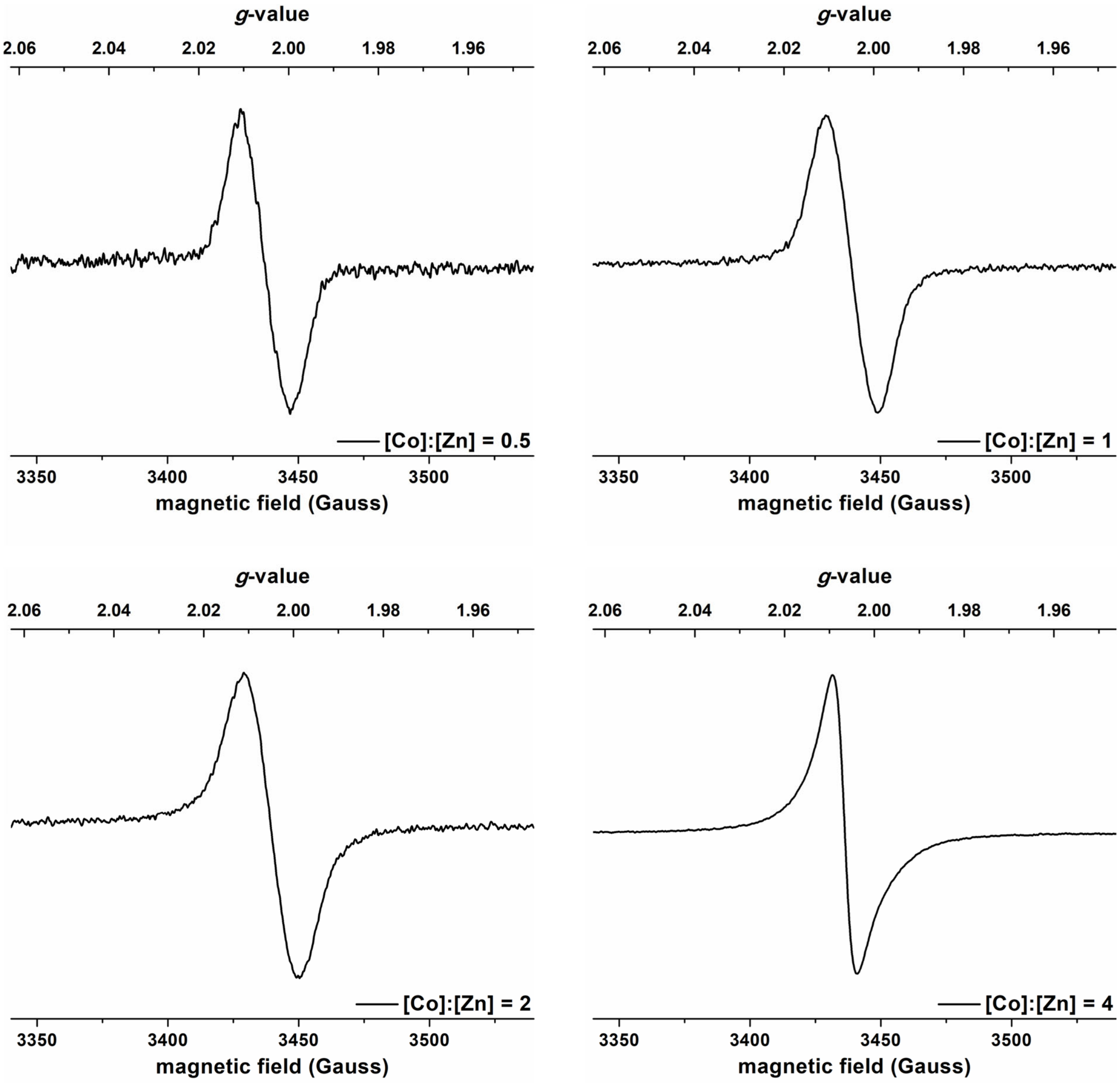

Figure S19. EPR spectra of **2** with different [Co]:[Zn] ratios in DCM ([Zn] = 10 mM).

Procedure:
Stock solutions of **2** (10.4 mg in 1 mL DCM) and Cp_2Co (9.5 mg in 500 µL DCM) were prepared in a glove box. Sample preparation consisted of transferring 250 µL of stock solution **2** to a vial, to which the appropriate amount of stock solution Cp_2Co was added, and the total volume was topped up with fresh DCM to 0.5 mL. The samples were transferred to an EPR tube, sealed with a cap, and wrapped with parafilm. The samples were removed from the glove box and measured on a Bruker EMX Nano X-band spectrometer. Double integration of the EPR spectra was performed using the Bruker Xenon software package and corrected for Q-value differences.

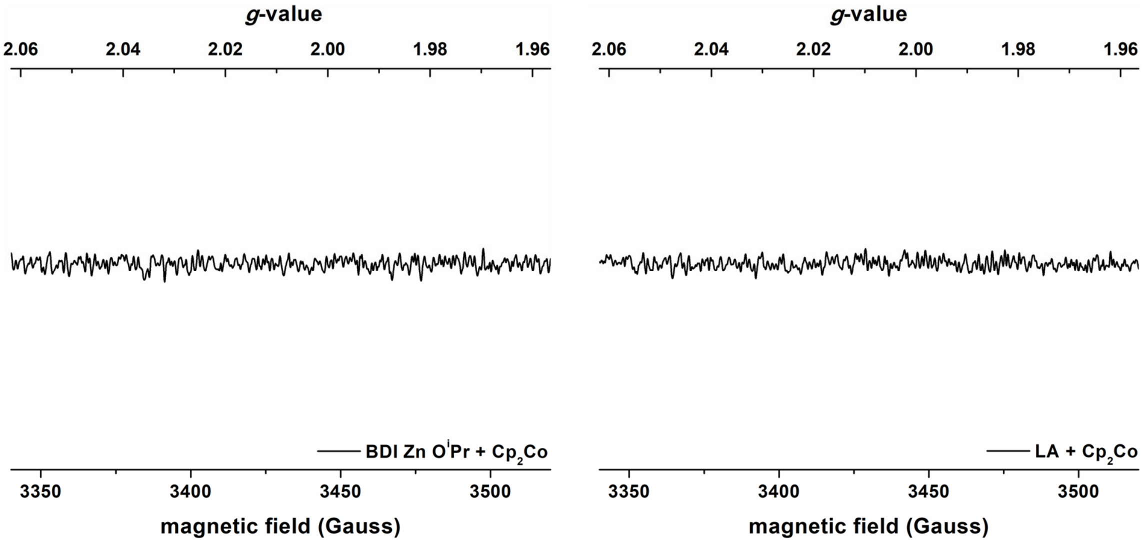

Figure S20. EPR spectra of (BDI)Zn(O*i*Pr) + Cp_2Co (left) and lactide + Cp_2Co (right) in DCM.

UV-Vis Spectroscopy

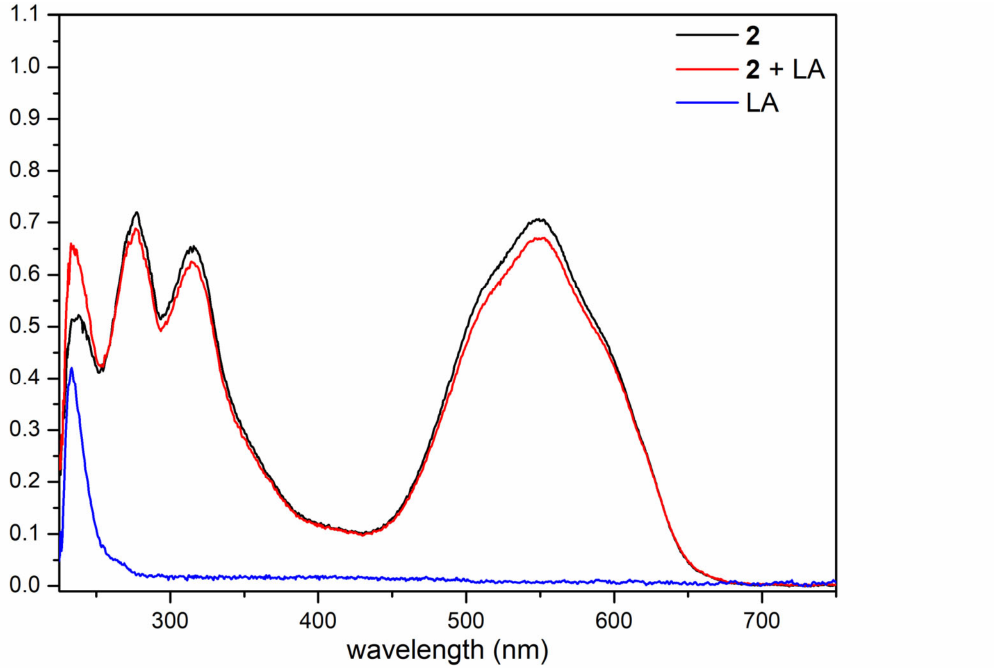

Figure S21. Absorption spectra for **2** (black), lactide (blue) and **2** + lactide (red) in DCM. Addition of lactide to **2** does not change the absorption spectrum (slightly lower intensity due to dilution). [**2**] ≈ 2.0 x 10^{-5} M, [**2**]:[LA] = 120.

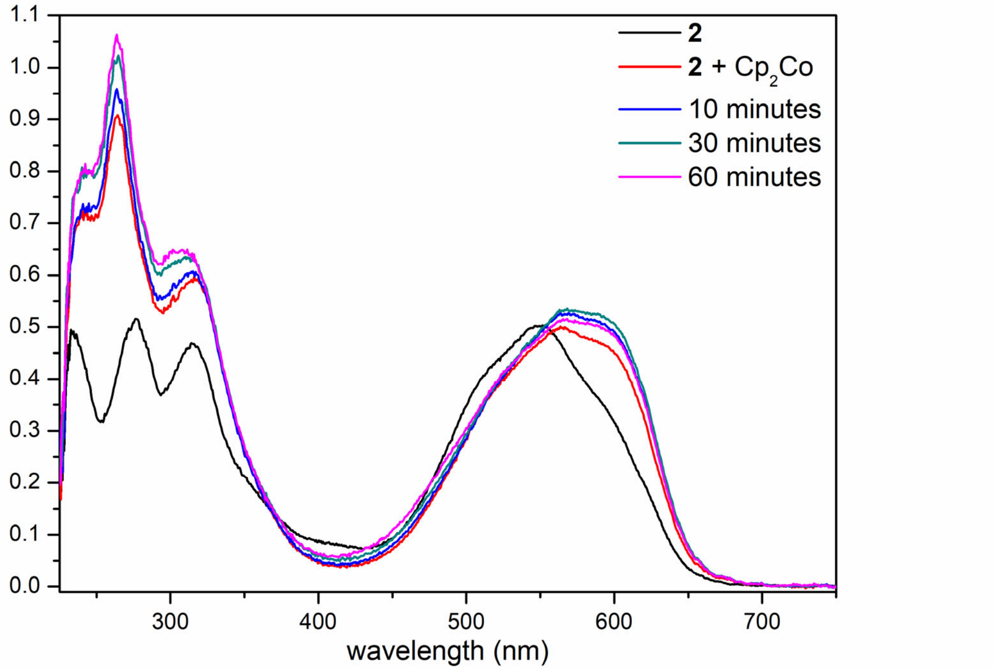

Figure S22. Absorption spectra of **2** (black), with 1 eq. of Cp_2Co immediately after addition (red), 10 minutes after addition (blue), 30 minutes after addition (teal), 60 minutes after addition (pink). [**2**] ≈ 1.5 x 10^{-5} M, [**2**]:[Cp_2Co] ≈ 1, spectra measured in DCM.

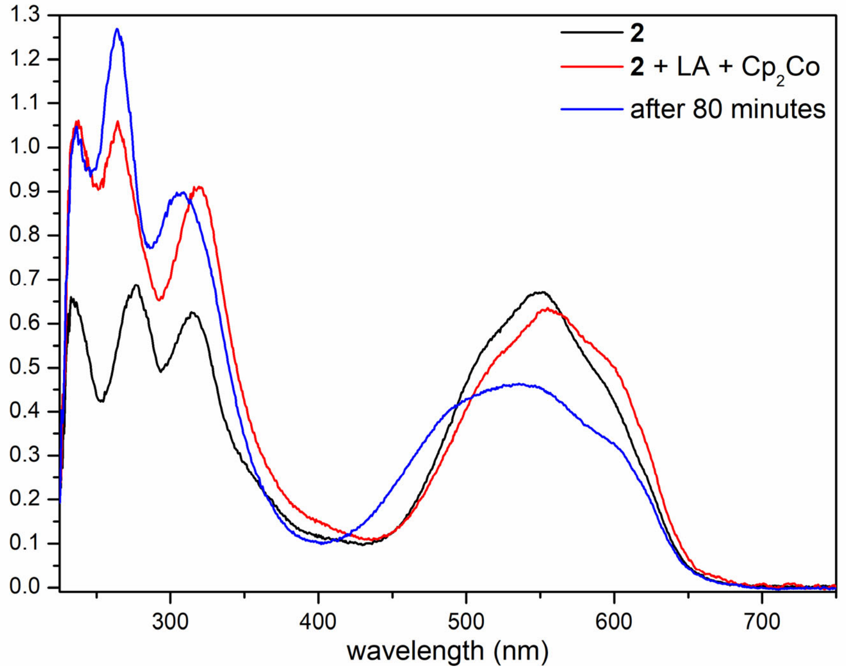

Figure S23. Absorption spectra for **2** (black) with Cp_2Co in the presence of lactide, taken shortly after addition of Cp_2Co (red) and 80 minutes after addition (blue). [**2**] ≈ 2.0 x 10^{-5} M, [**2**]:[LA] ≈ 120, [Zn]:[Cp_2Co] ≈ 1, spectra measured in DCM.

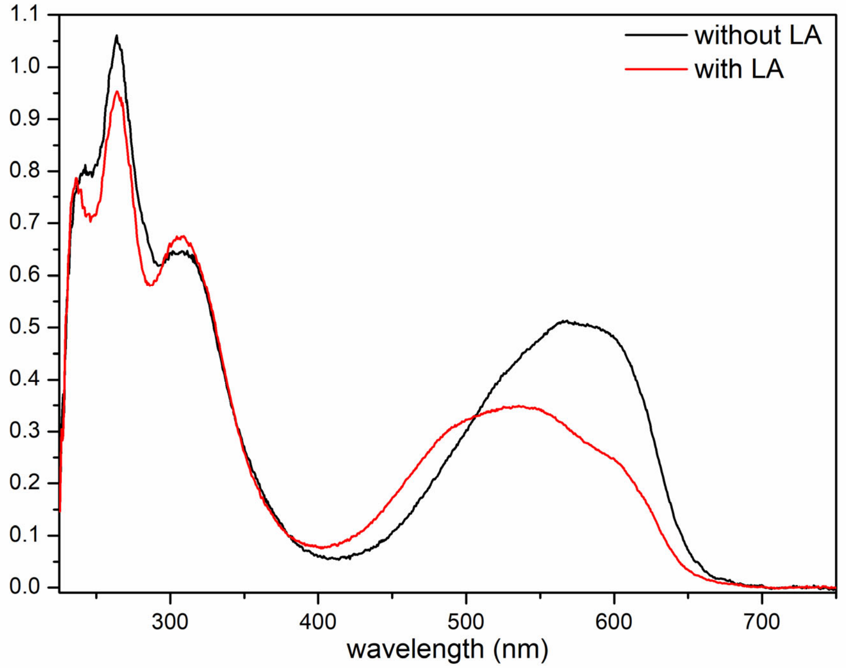

Figure S24. Absorption spectra for **2**/Cp_2Co, with lactide (red) and without lactide (black) present, recorded in DCM.

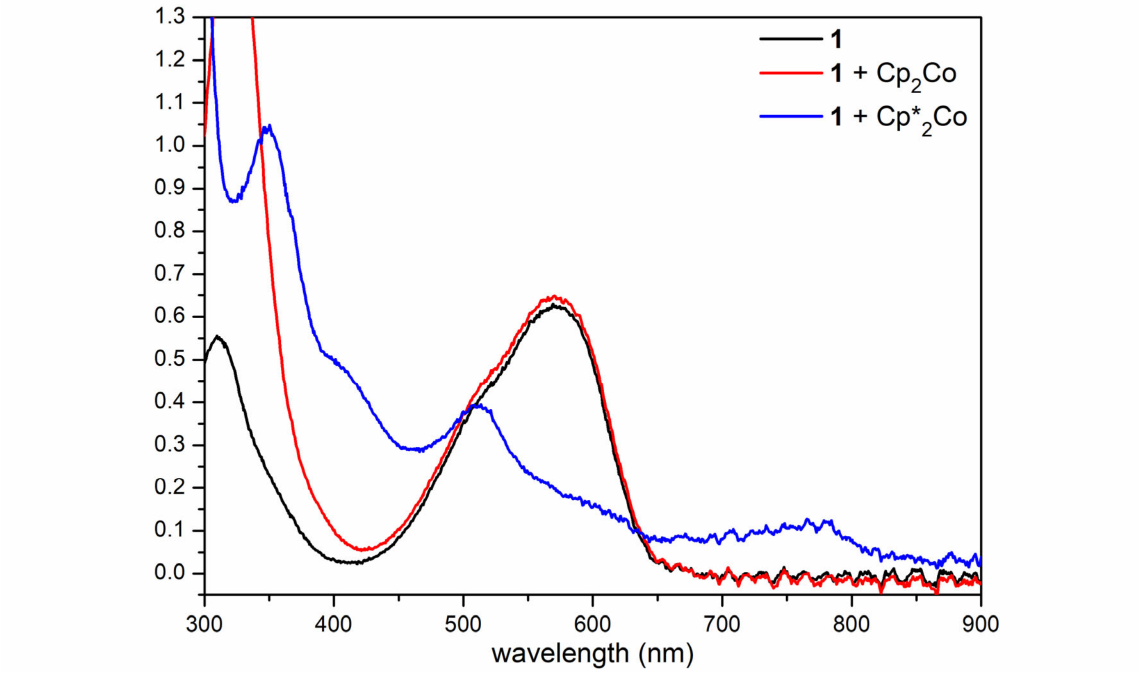

Figure S25. Absorption spectra for **1** (black), **1** + 1 eq. Cp_2Co (red) and **1** + 1 eq. Cp^*_2Co (blue). [**1**] ≈ 2.5 x 10^{-5} M, spectra measured in THF.

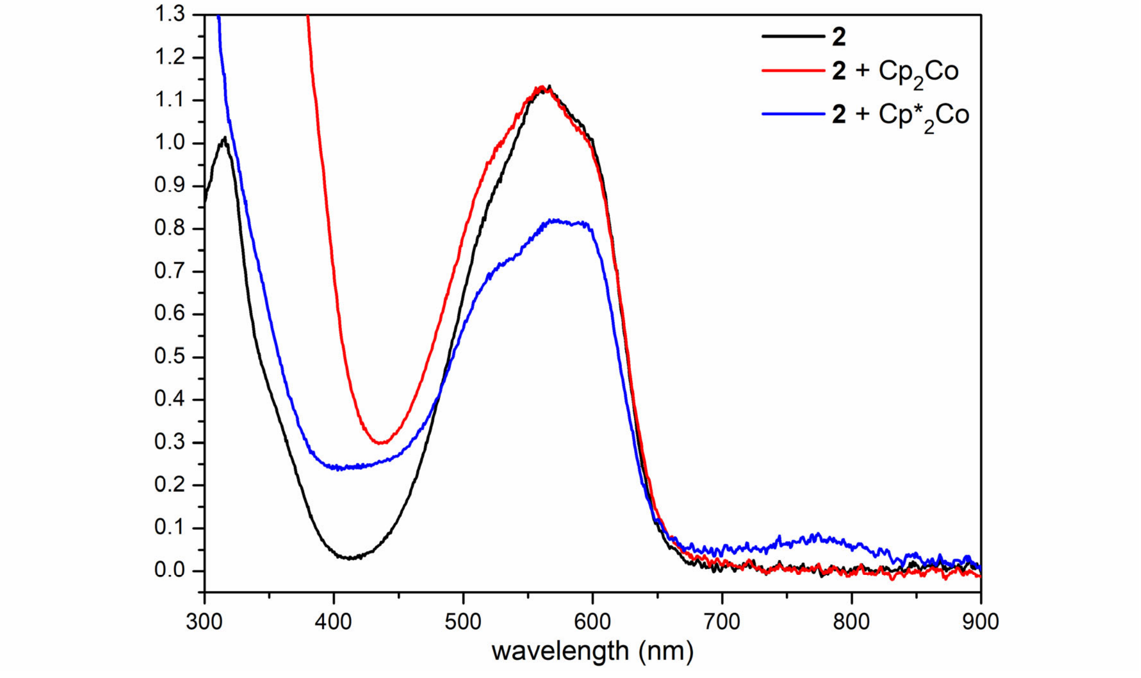

Figure S26. Absorption spectra for **2** (black), **2** + 1 eq. Cp_2Co (red) and **2** + 1 eq. Cp^*_2Co (blue). [**2**] ≈ 2.5 x 10^{-5} M, spectra measured in THF.

Direct Injection Mass Spectrometry

Procedure:

A polymerization reaction was initiated ([LA]:[Zn] = 10, [Co]:[Zn] = 1) in the glovebox. An aliquot from the reaction mixture was taken using a microsyringe, which was subsequently capped with a rubber stopper. The microsyringe was attached to the mass spectrometer (no column) and the aliquot was directly injected into the ionization chamber. Spectra were measured in negative ion mode and summed over 30 seconds of measuring. The resulting spectrum shows a well-defined envelope, starting at 377.03 Da, with increments of 72 Da. This is in agreement with growing polymer chain attached to a (formazanate)zinc fragment

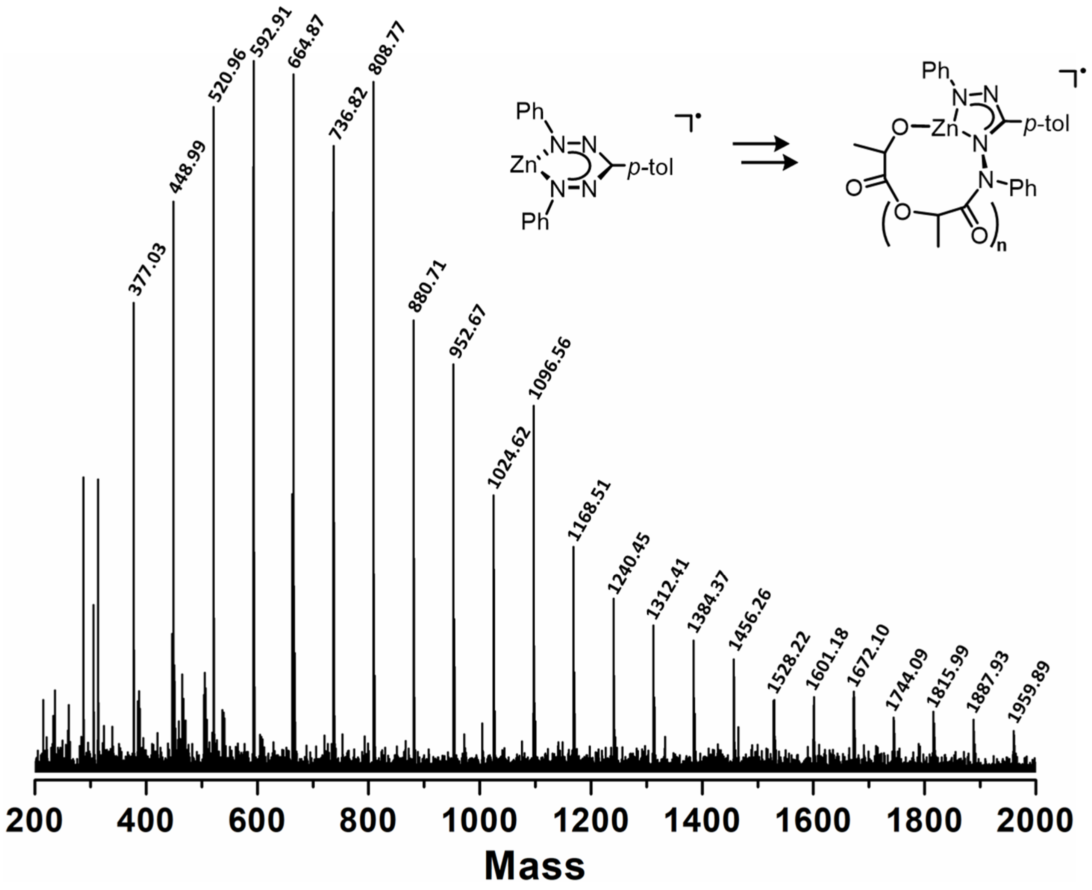

Figure S27. Mass spectrum obtained after direct injection of a “living” polymerization sample ([LA]:[Zn] = 10, [Co]:[Zn] = 1).

Characterization of the Resulting Polymers

Gel Permeation Chromatography

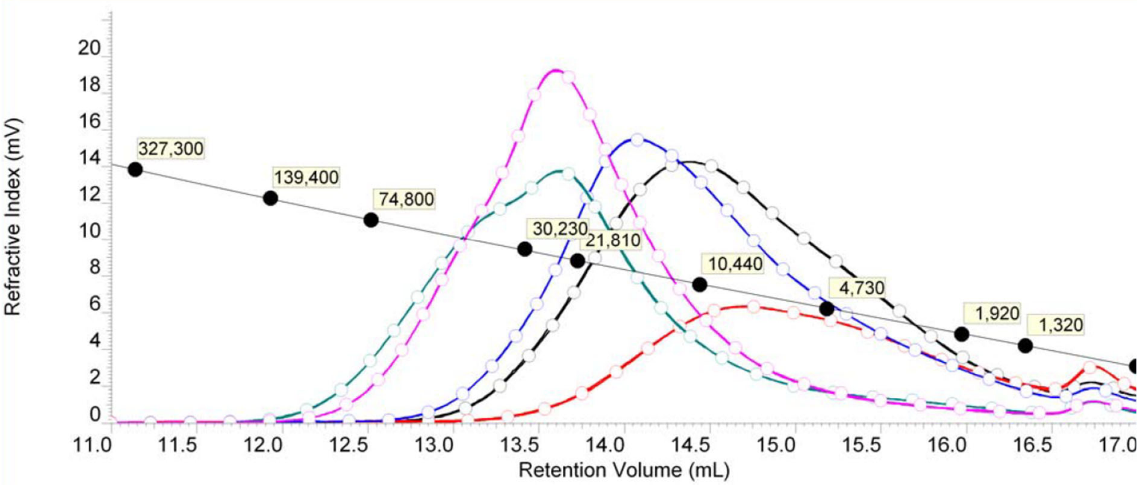

Figure S28. Representative GPC traces for PLA obtained using **2**. Sample measured in THF at 35°C and reported as absolute molecular weight (Table 1).

^{1}H NMR Spectrum

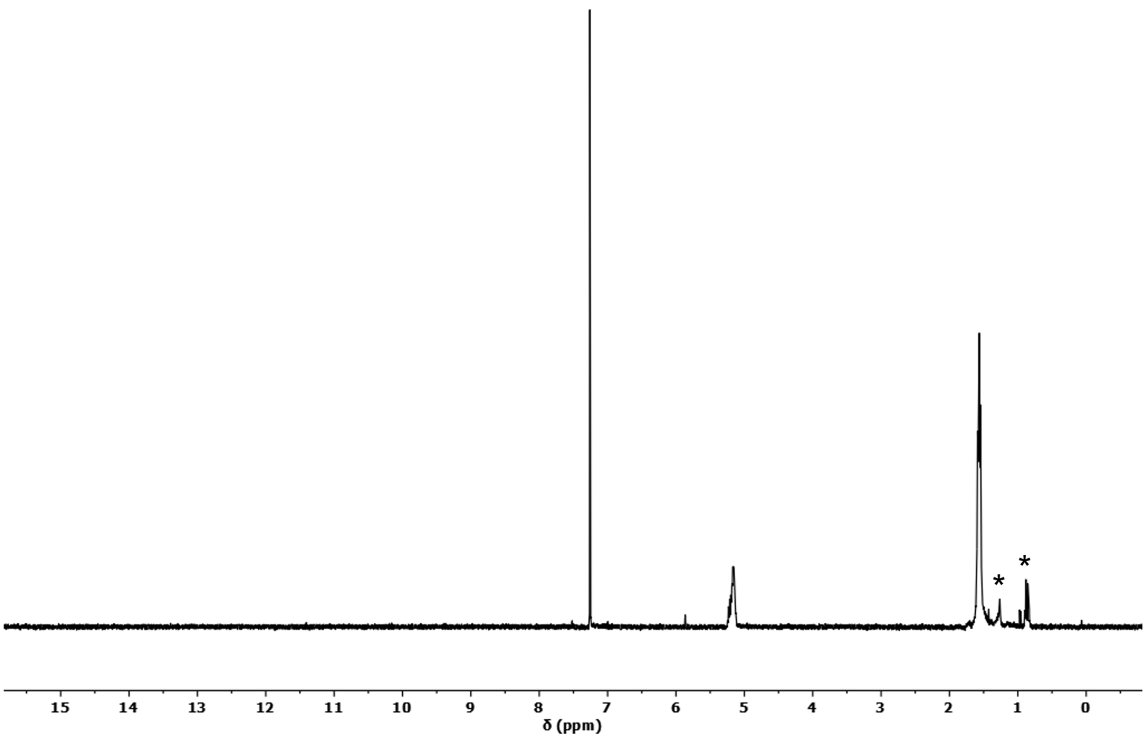

Figure S29. ^{1}H NMR spectrum of produced from the reaction of *rac*-lactide with **2**/Cp_2Co ([LA]:[Zn] = 50, [Co]:[Zn] = 1) ($CDCl_3$, 25 °C, 400 MHz). * denotes residual hexane from precipitation of the polymer.

MALDI-ToF

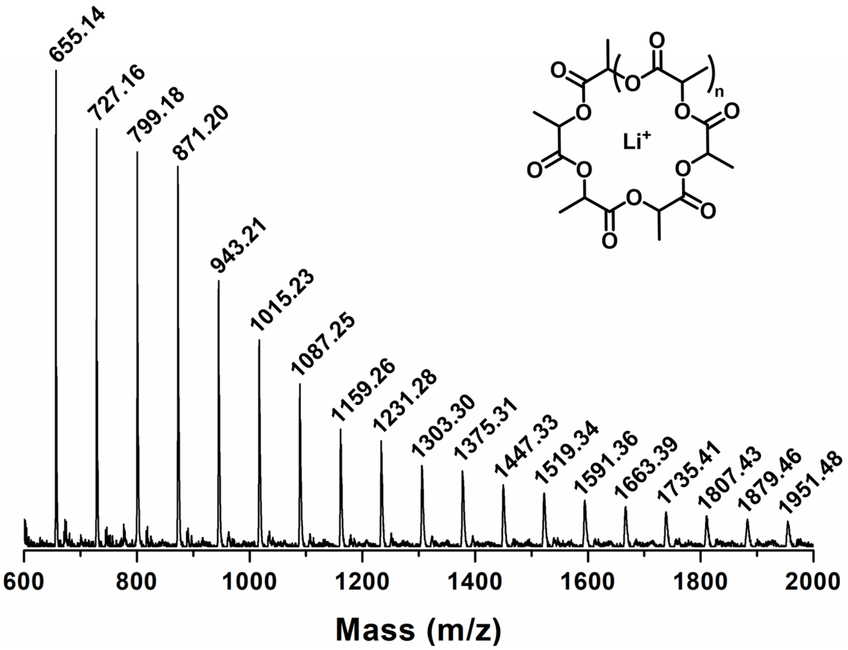

Figure S30. MALDI-ToF spectrum of polymer produced from the reaction of *rac*-lactide with **2**/Cp_2Co ([LA]:[Zn] = 50, [Co]:[Zn] = 1).

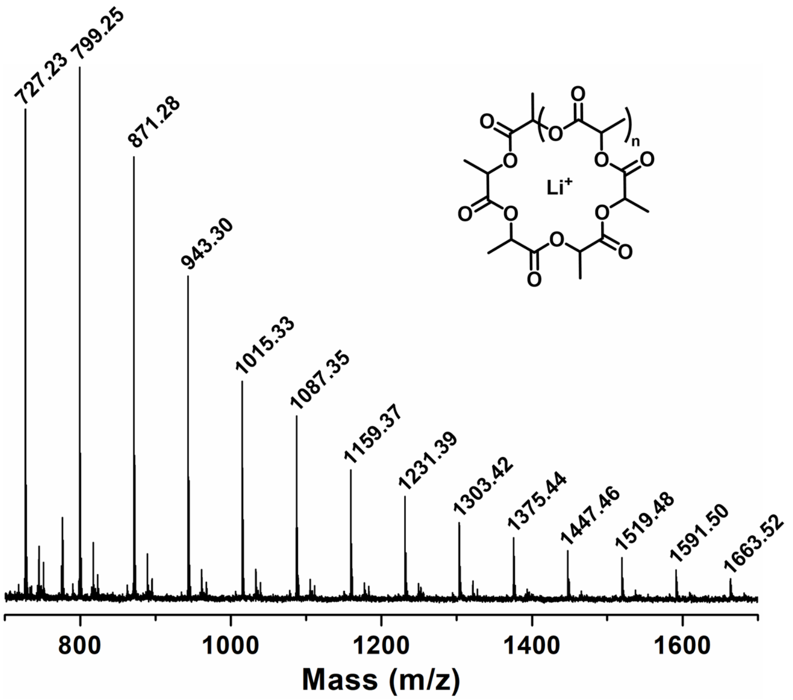

Figure S31. MALDI-ToF spectrum of polymer produced from the reaction of *rac*-lactide with **2**/Cp_2Co ([LA]:[Zn] = 10, [Co]:[Zn] = 1).

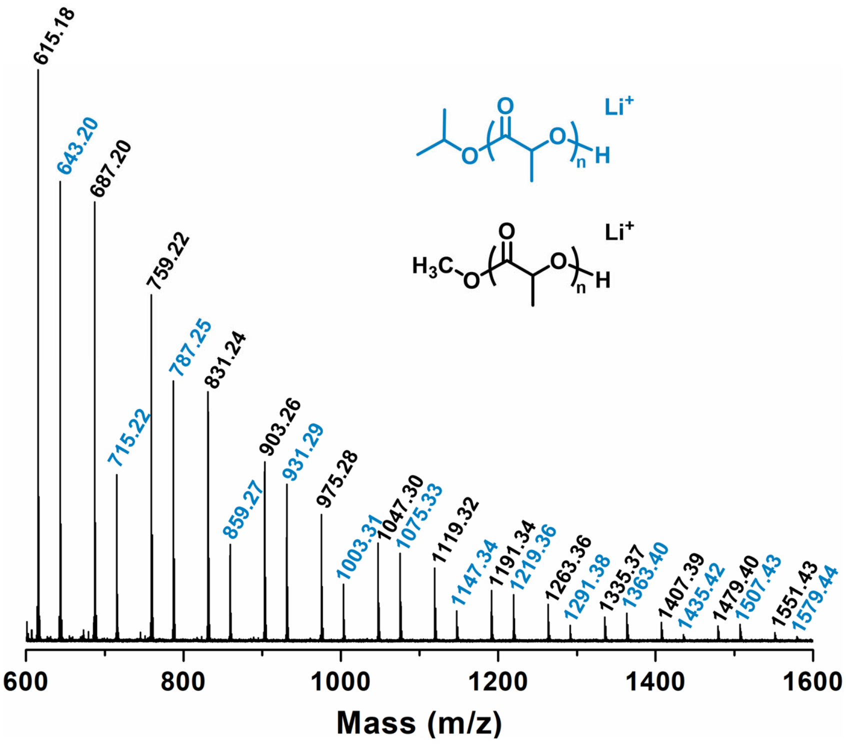

Figure S32. MALDI-ToF spectrum of polymer produced from the reaction of *rac*-lactide with **([BDI]ZnO^{i}Pr)$_2$** ([LA]:[Zn] = 10, [Co]:[Zn] = 1).

The polymerization of *rac*-lactide with the ([BDI]ZnO^{i}Pr)$_2$ catalyst, as described by Coates *et al.*[15], was performed as a control experiment. The MALDI-ToF spectrum of the product obtained from polymerization showed a clearly identifiable envelope corresponding to the linear polymer with isopropyl initiator (next to an envelope corresponding to polymer with methanol as end group, produced during the quenching of the polymerization with methanol). This result substantiates that the formation of the cyclic polymer using our system does not come from a measurement error.

Mn versus conversion plot

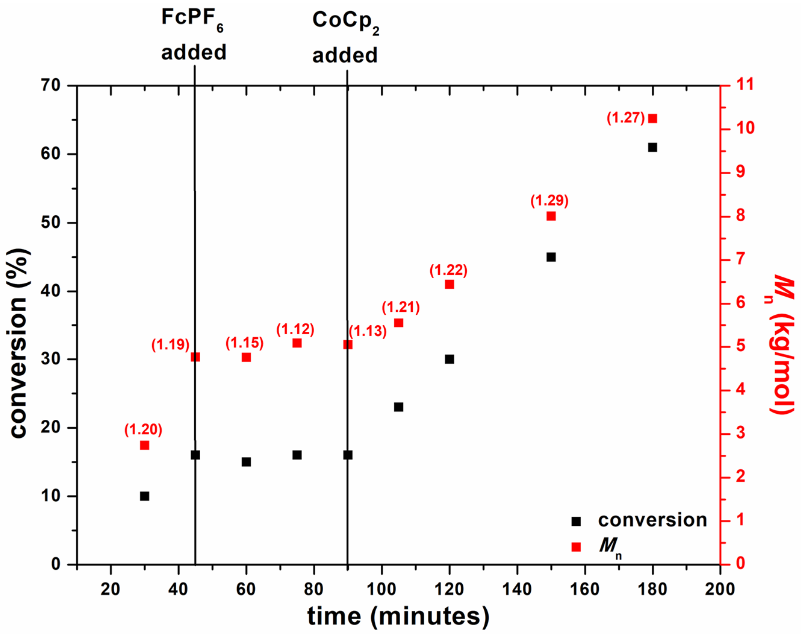

Figure S33. Polymerization of *rac*-lactide by complex **2** ($[LA]_0/[Zn]_0 = 50$, $[LA]_0 = 0.5$ M, [Co]/[Zn] = 1), showing conversion of lactide (black squares), molecular weight (red squares) and dispersity (in parentheses) over time, demonstrating the linear relationship between molecular weight and conversion. $FcPF_6$ added after 45 minutes, Cp_2Co added after 90 minutes.

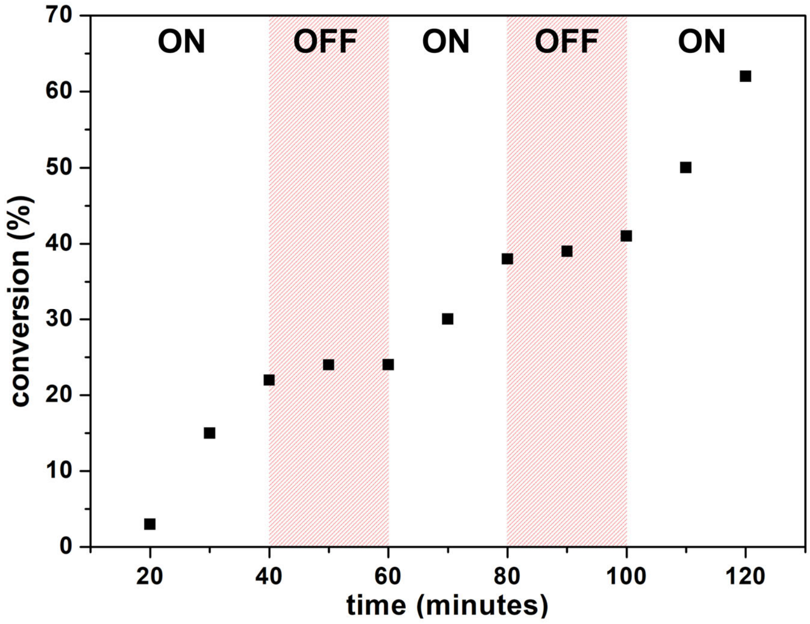

Figure S34. Conversion of lactide ($[LA]_0/[Zn]_0 = 50$, $[LA]_0 = 0.5$ M, [Co]/[Zn] = 1) with multiple switches between catalysts states ($FcPF_6$ added after 40 and 80 minutes; Cp_2Co added at the start and after 60 and 100 minutes).

References

(1) Chang, M.-C. Formazanate as a Redox-Active, Structurally Versatile Ligand Platform, University of Groningen, 2016.
(2) de Vries, F.; Travieso-Puente, R.; Roewen, P.; Otten, E. Three-Coordinate Zinc Methyl Complexes with Sterically Demanding Formazanate Ligands *Organometallics* **2021**, *40*, 63-71.
(3) Gregson, C. K. A.; Gibson, V. C.; Long, N. J.; Marshall, E. L.; Oxford, P. J.; White, A. J. P. Redox Control within Single-Site Polymerization Catalysts *J. Am. Chem. Soc.* **2006**, *128*, 7410-7411.
(4) Broderick, E. M.; Guo, N.; Wu, T.; Vogel, C. S.; Xu, C.; Sutter, J.; Miller, J. T.; Meyer, K.; Cantat, T.; Diaconescu, P. L. Redox control of a polymerization catalyst by changing the oxidation state of the metal center *Chem. Commun.* **2011**, *47*, 9897-9899.
(5) Wang, X.; Thevenon, A.; Brosmer, J. L.; Yu, I.; Khan, S. I.; Mehrkhodavandi, P.; Diaconescu, P. L. Redox Control of Group 4 Metal Ring-Opening Polymerization Activity toward l-Lactide and ε-Caprolactone *J. Am. Chem. Soc.* **2014**, *136*, 11264-11267.
(6) Hern, Z. C.; Quan, S. M.; Dai, R.; Lai, A.; Wang, Y.; Liu, C.; Diaconescu, P. L. ABC and ABAB Block Copolymers by Electrochemically Controlled Ring-Opening Polymerization *J. Am. Chem. Soc.* **2021**, *143*, 19802-19808.
(7) Biernesser, A. B.; Li, B.; Byers, J. A. Redox-controlled polymerization of lactide catalyzed by bis(imino)pyridine iron bis(alkoxide) complexes *J. Am. Chem. Soc.* **2013**, *135*, 16553-16560.
(8) Qi, M.; Dong, Q.; Wang, D.; Byers, J. A. Electrochemically Switchable Ring-Opening Polymerization of Lactide and Cyclohexene Oxide *J. Am. Chem. Soc.* **2018**, *140*, 5686-5690.
(9) Brown, L. A.; Rhinehart, J. L.; Long, B. K. Effects of Ferrocenyl Proximity and Monomer Presence during Oxidation for the Redox-Switchable Polymerization of l-Lactide *ACS Catalysis* **2015**, *5*, 6057-6060.
(10) Doerr, A. M.; Burroughs, J. M.; Legaux, N. M.; Long, B. K. Redox-switchable ring-opening polymerization by tridentate ONN-type titanium and zirconium catalysts *Catal. Sci. Technol.* **2020**, *10*, 6501-6510.
(11) Bruker., *Apex3, SAINT and SADABS*, **2016**, Bruker AXS Inc.: Madison, WI, USA.
(12) Sheldrick, G. A short history of SHELX *Acta Crystallogr. A* **2008**, *64*, 112-122.
(13) Sheldrick, G. Crystal structure refinement with SHELXL *Acta Crystallogr. C* **2015**, *71*, 3-8.
(14) Spek, A. PLATON SQUEEZE: a tool for the calculation of the disordered solvent contribution to the calculated structure factors *Acta Crystallographica Section C* **2015**, *71*, 9-18.
(15) Chamberlain, B. M.; Cheng, M.; Moore, D. R.; Ovitt, T. M.; Lobkovsky, E. B.; Coates, G. W. Polymerization of Lactide with Zinc and Magnesium β-Diiminate Complexes: Stereocontrol and Mechanism *J. Am. Chem. Soc.* **2001**, *123*, 3229-3238.
